# From Waste to Resource: Upcycling Spent Coffee Grounds into Biochar Functionalized with CaFe_2_O_4_ for Phosphate Removal

**DOI:** 10.1021/acsomega.6c04188

**Published:** 2026-09-25

**Authors:** Yago Neco Teixeira, Marcella Araujo Macedo, André Vinicius Lopes Marques, Daniel Bernardes Silva, Jorge Marcell Coelho Menezes, Thiago Mielle Brito Ferreira Oliveira, Raimundo Nonato Pereira Teixeira, Adonay Rodrigues Loiola, Ronaldo Ferreira do Nascimento, Maria Gilnara Lima Bandeira, Mônica Belém Rodrigues, João Maria Soares, Meirielle Marques de Góis, Francisco José de Paula Filho

**Affiliations:** † Biological Chemistry Department, Regional University of Cariri, R. Cel. Antonio Luis, 1161, Crato, Ceará 63105-000, Brazil; ‡ Science and Technology Center, 28121 Federal University of Cariri, Av. Ten. Raimundo Rocha, 1639, Juazeiro do Norte, Ceará 63048-080, Brazil; § Department of Organic and Inorganic Chemistry, Federal University of Ceará, Fortaleza, Ceará 60440-900, Brazil; ∥ Analytical and Physical Chemistry Department, Federal University of Ceará, Fortaleza, Ceará 60356-000, Brazil; ⊥ Materials Characterization Laboratory, Federal University of Ceará, Russas, Ceará 62900-000, Brazil; # Physics Department, State University of Rio Grande do Norte, Mossoró, Rio Grande do Norte 59625-620, Brazil

## Abstract

Eutrophication caused by excess phosphorus in water bodies represents a significant environmental challenge, requiring efficient, selective, and sustainable technologies for its mitigation. In this study, a magnetic biochar (BiMag) was developed through the valorization of residual coffee grounds and functionalized with calcium ferrite (CaFe_2_O_4_) for phosphate removal from aqueous media. The synthesis of the composite was optimized using response surface methodology (Box–Behnken), evaluating the effects of precipitation time, CaCl_2_ volume, temperature, and calcination time. The optimal condition obtained consisted of 102 min of precipitation at 20 °C, 18 mL of CaCl_2_ (3 mol/L), calcination at 400 °C for 120 min, with 11 mL of FeCl_3_ (3 mol/L). Material characterization confirmed the incorporation of CaFe_2_O_4_, a highly porous rough morphology, and superparamagnetic behavior. Adsorption studies indicated high efficiency and selectivity for phosphate, with minimal influence of pH and the presence of competing anions. Adsorption kinetics were well described by the Elovich and pseudo-second-order (PSO) models, while equilibrium data were adequately represented by the Sips isotherm, with a maximum phosphate adsorption capacity of 97 mg/g. The adsorption process was spontaneous and endothermic, involving combined mechanisms of surface complexation, ligand exchange, and precipitation of calcium and iron phosphates. These results demonstrate that BiMag is an efficient, selective, and regenerable adsorbent with high potential for application in wastewater treatment and sustainable phosphorus recovery, in alignment with the principles of the circular economy.

## Graphical abstract

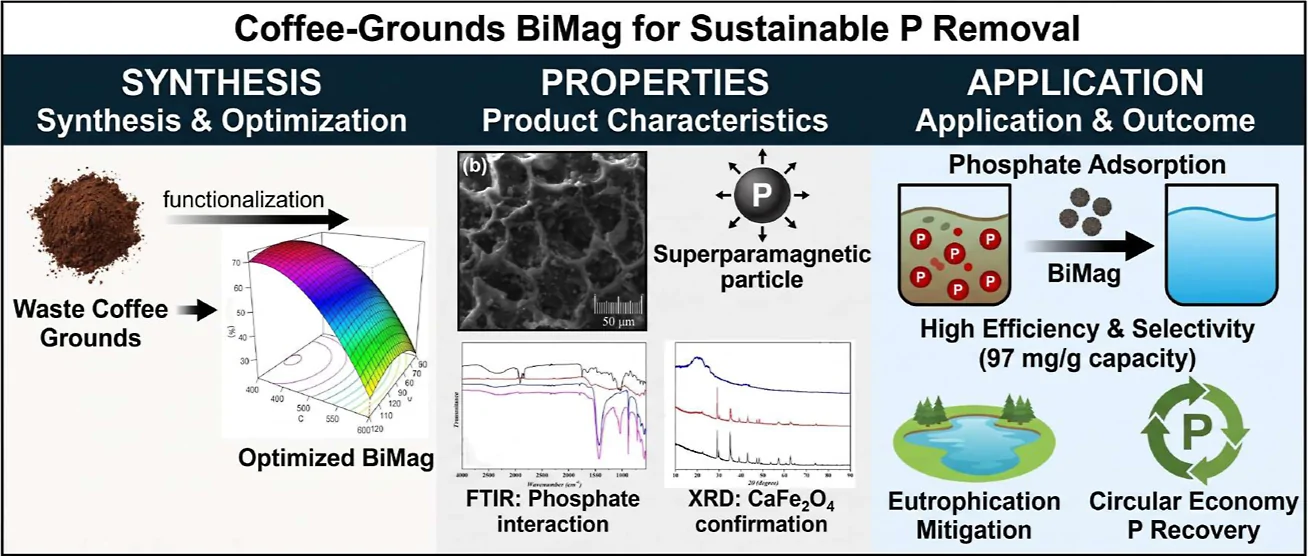

## 1 Introduction

Eutrophication, resulting from the excessive input of phosphorus—especially in the form of phosphate—into water bodies, constitutes one of the major contemporary challenges to water quality on a global scale. Among the various impacts associated with the excess of this nutrient, uncontrolled algal proliferation stands out, leading to a reduction in dissolved oxygen and, consequently, compromising the ecological balance and ecosystem services of aquatic environments.

In recent years, literature reviews have highlighted a substantial increase in research focused on the removal and recovery of phosphorus from effluents and eutrophicated waters, both as an environmental mitigation strategy and as an alternative for the reuse of a finite resource essential for fertilizer production.^1–7^

Phosphate concentrations in wastewater vary according to their source. In raw wastewater, total phosphorus typically ranges from 5 to 15 mg/L, decreasing to 2–6 mg/L after conventional treatment due to the limited phosphorus removal achieved by biological processes. In effluents from the food processing industry and slaughterhouses, phosphate concentrations generally range from 10 to 30 mg/L or higher, whereas effluents from fertilizer and chemical industries may exceed 100 mg/L.^1,5,6^

Several approaches have been investigated for phosphate removal from aqueous media, which can be grouped into three main categories: chemical methods, such as precipitation and adsorption;^8,9^ physical methods, such as microfiltration and reverse osmosis;^10,11^ and biological methods, such as assimilation.^12^

Among these alternatives, adsorption stands out by offering significant advantages, such as low operational cost, simplicity of application, and high efficiency in contaminant removal. Moreover, this process enables phosphorus recovery in potentially reusable forms, allowing the production of higher value-added inputs, such as fertilizers and agricultural amendments, thereby reinforcing its strategic role in circular economy practices and in the promotion of environmental sustainability.

Low-cost, environmentally friendly, and easily produced adsorbents for phosphate removal from water have gained increasing attention in recent years. Among these materials are synthetic composites,^13–16^ chitosan,^7^ metal–organic frameworks,^17^ oxides and (hydr)­oxides,^3,18^ ashes,^19^ and biochars.^20–23^

Although biochars derived from agricultural or urban residues offer advantages such as low cost, wide availability, and high specific surface area, unmodified biochars often exhibit little to no adsorption capacity and reduced selectivity for phosphate.^24–26^ Consequently, there is growing interest in modified biochars.

Recent literature has focused on the chemical or physical modification of biochar through the incorporation of metals, such as iron, magnesium, calcium, and manganese, or metal oxides, with the aim of increasing active complexation sites, ion-exchange capacity, and the formation of precipitated phosphate phases.^27–29^

Iron- or calcium-enriched biochars exhibit superior performance compared to unmodified biochars. The presence of mixed calcium and iron phases, such as calcium ferrite (CaFe_2_O_4_), can significantly enhance adsorption capacity. However, important gaps remain, including the very limited number of studies exploring the use of CaFe_2_O_4_ supported on a biochar matrix, the controlled formation of the ferrite phase (Ca–Fe composition, calcination temperature), as well as regenerability and feasibility for phosphate recovery.

Compared with conventional Fe–Ca binary oxides or layered double hydroxides (LDHs), biochar/calcium ferrite composites exhibit significant structural, operational, and economic advantages. The carbonaceous biochar support promotes greater dispersion of the Ca and Fe metallic phases, preventing nanoparticle agglomeration and significantly increasing the accessibility of active sites for interaction with phosphate species. Biochar-supported materials exhibit higher surface area, pore volume, and intraparticle diffusion than pure metal oxides, thereby favoring higher adsorption capacities and improved removal kinetics.^20,30,31^

Furthermore, biochar-supported materials exhibit greater oxide dispersion, higher accessibility to active sites, and improved mass transport within the adsorbent structure. Isolated metal oxides frequently exhibit low porosity and a tendency toward sintering or aggregation during synthesis, which reduces adsorption efficiency. The biochar matrix minimizes these effects and enhances system stability.^20^

The use of spent coffee grounds as a precursor for the production of adsorbent biochar presents significant environmental and technological relevance due to the large global generation of this residue and the impacts associated with its improper disposal. Coffee is among the most widely consumed agricultural commodities worldwide, with an annual production exceeding 10 million tons of beans. Consequently, large quantities of solid waste are generated throughout the production and consumption chain, particularly spent coffee grounds, which are mainly produced in coffee shops, food industries, and domestic beverage preparation.^32^

The processing of one metric ton of green coffee generates approximately 650 kg of spent coffee grounds, whereas instant coffee production generates about 2 kg of wet spent coffee grounds per kilogram of final product. Traditionally, this residue is disposed of in landfills or through incineration, practices that contribute to greenhouse gas emissions, odor generation, and the formation of acidic leachates capable of contaminating soil and water bodies. Furthermore, the release of compounds such as caffeine, chlorogenic acids, and tannins may cause ecotoxicological effects, while the direct application of spent coffee grounds to soil may impair seed germination, plant growth, and microbial activity.^32^

In this context, the conversion of spent coffee grounds into biochar represents a strategy aligned with the principles of circular economy and agro-industrial waste valorization. In addition to reducing environmental liabilities, the resulting biochar exhibits a stable carbonaceous structure, oxygen-containing functional groups, and potential for surface modification aimed at environmental applications, including the removal of aqueous contaminants.^33^

Although several studies have investigated biochars for phosphate adsorption, as well as metal-modified biochars, the novelty of the present work lies primarily in the specific combination of spent coffee grounds and calcium ferrite (CaFe_2_O_4_), as well as in the synergistic mechanism associated with the simultaneous presence of calcium and iron species supported on a residual carbonaceous matrix.

Most studies reported in the literature focus on biochars modified with simple iron salts, isolated oxides, or layered double hydroxides (LDHs). However, only a limited number of studies have specifically explored calcium ferrites as the active phase supported on biochar derived from spent coffee grounds. This distinction is relevant because calcium ferrites exhibit chemical properties different from those of conventional oxides, potentially providing both ferric sites for surface complexation and calcium species capable of promoting the precipitation of sparingly soluble phosphates. Thus, the developed material combines multiple removal mechanisms within a single adsorbent structure.

The development and evaluation of a composite in which CaFe_2_O_4_ is supported on biochar can simultaneously address environmental and resource circularity demands. Accordingly, the present study aimed to develop this composite. This approach seeks to enhance phosphate removal from effluents and eutrophicated waters through the use of a low-cost, renewable material, while also exploring synthesis routes capable of controlling the formation of Ca–Fe phases on the porous biochar surface, thereby optimizing adsorption capacity and selectivity.

## 2 Materials and Methods

### 2.1 BiMag Synthesis and Design of Experiments (DoE)

Spent coffee grounds were collected from households in the city of Juazeiro do Norte, Brazil. After collection, they were washed with deionized water, carefully separated from other solid residues, dried at 105 °C for 2 h, and sieved through an 40-mesh sieve (<400 μm).

For the synthesis of BiMag, 5 g of sieved coffee grounds were used per sample. The material was initially placed in a 100 mL beaker, followed by the addition of 20 mL of H_2_O, a CaCl_2_ solution (3 mol/L), 9 mL of FeCl_3_ (3 mol/L), and 30 mL of NaOH (5 mol/L). The volume of CaCl_2_ was adjusted according to the design of experiments (DoE). The mixture was kept under constant stirring at 20 °C on a magnetic stirrer, with the precipitation time—here defined as the duration of this process—also varied according to the DoE.

Next, the mixture (precipitate plus coffee grounds) was centrifuged at 3000 rpm for 5 min to separate the liquid phase. The obtained material was then dried in an oven at 110 °C for 2 h and subsequently subjected to calcination in a muffle furnace, with temperature and time adjusted according to the design of experiments (DoE)—the step responsible for BiMag formation. After calcination, the BiMag was washed with a 0.1 mol/L HCl solution until neutral pH (∼7) was reached.

For the optimization of BiMag synthesis parameters, the Box–Behnken model was chosen due to its distinct advantages: it avoids experimental runs at extreme levels of all factors and provides an efficient capability to estimate first- and second-order coefficients.^34^

Four different factors were considered—precipitation time (A), CaCl_2_ solution volume (B), calcination temperature (C), and calcination time (D)—with each factor consisting of three levels: low (−1), high (+1), and central (0) for phosphate removal efficiency.

For precipitation time, the following values were used: 60, 90, and 120 min. For the volume of CaCl_2_ solution (3 mol/L), the values were 7, 14.5, and 22 mL. For calcination temperature, the values were 400, 500, and 600 °C. For calcination time, the values were 60, 120, and 180 min. A total of 27 experiments were conducted to fit the polynomial model (eq S1). All equations used in this work are provided in the Supporting Information.

The selection of the factors evaluated in this study was based on their direct influence on the structural and surface properties of calcium ferrite-based materials—such as crystallinity, surface area, and the presence of functional groups—which significantly affect phosphate adsorption capacity.

The precipitation time and the volume of the CaCl_2_ solution were selected based on their influence on nucleation and crystal growth, which affect particle size, specific surface area, and the distribution between amorphous and crystalline phases. The calcination temperature was chosen because of its critical role in promoting structural transformations, including increased crystallinity and the formation of more stable and active phases. Calcination time was included as an experimental factor due to its influence on phase evolution and the extent of thermal transformations.^18^

All results were analyzed by ANOVA using R software, version 4.2.2, and RStudio, version 2023.12.1. Once the optimal values for the four factors were identified, they were applied in the subsequent syntheses. Additionally, the effects of precipitation temperature and FeCl_3_ solution volume (3 mol/L) were evaluated separately to optimize all synthesis parameters.

### 2.2 BiMag Characterization

The microstructure, morphology, and surface composition of unmodified biochar (BiNat), BiMag, and BiMag after phosphate adsorption (BiMag + P) were characterized by SEM–EDS, while the functional groups present on the surface were identified by FTIR and Raman spectroscopy. The crystalline structure was determined by XRD in a 2θ range of 10°–90°, using Kα radiation (λ = 1.54178 Å). The average crystallite size was estimated from the broadening of diffraction peaks using the Scherrer equation (eq S2). N_2_ gas adsorption–desorption isotherms were obtained at −196 °C over a wide relative pressure range from 0.01 to 0.995 atm with a volumetric adsorption analyzer Micromeritics ASAP 2020 plus. The sample was outgassed at 300 °C for 12 h prior to the measurements. The specific area was calculated by Brunauer–Emmett–Teller (BET) method and the pore size distribution was calculated by BJH. The magnetic properties of BiMag were investigated by vibrating sample magnetometry (VSM). The point of zero charge (pH_PZC_) of BiMag was determined using the solid addition method.^35^

### 2.3 Adsorption Experiments

Table S1 presents all values and parameters analyzed in the adsorption tests. BiMag was separated from the solutions using an external magnetic field after the completion of each experiment. The residual phosphate concentration in the samples was determined using the Murphy and Riley method,^36^ with a UV–VIS spectrophotometer (Shimadzu, UV 1800) at a fixed wavelength of 882 nm. Adsorption capacities were calculated using eqs S3 and S4, and removal efficiency was calculated using eq S5. For the anion coexistence experiments, the phosphate concentration was maintained at 10 mg/L, while the concentrations of the analyzed anions (Cl^–^, NO_3_^–^, SO_4_^2–^, HCO_3_^–^, and CO_3_^2–^) were varied. pH adjustments were made using HCl (0.1 mol/L) and NaOH (0.1 mol/L) solutions.

### 2.4 Modeling of Adsorption Kinetics, Isotherms, and Thermodynamics

Kinetic modeling was performed using the Pseudo-First-Order (PFO—eq S6), Pseudo-Second-Order (PSO—eq S7), and Elovich (eq S8) models. For equilibrium modeling, the Langmuir (eq S9), Freundlich (eq S10), Sips (eq S11), and Dubinin–Radushkevich (DR—eq S12) isotherm models were applied, from which the average adsorption energy was estimated (eq S13). Thermodynamic modeling of adsorption was conducted based on the Van’t Hoff equation (eq S14), with the Gibbs free energy calculated using eq S15.

### 2.5 Adsorption–Desorption Cycles

Adsorption–desorption cycles were analyzed to evaluate the regenerability of BiMag. NaOH solutions at different concentrations were used as eluents: NaOH (0.1 mol/L), NaOH (0.5 mol/L), and NaOH (1 mol/L). This concentration range was selected for the eluents to prevent structural damage to the BiMag material while maintaining the economic and environmental viability of the process. The desorption efficiency of the eluents was calculated using eq S16. After each adsorption/desorption test, BiMag was washed and centrifuged at 3000 rpm for 20 min to minimize mass loss between cycles.

## 3 Results and Discussion

### 3.1 Optimization Using Response Surface Methodology

The phosphate removal results under different synthesis parameters are presented in Table S2. Removal efficiencies ranged from 11.17% to 72.28%. The synthesis condition that yielded the lowest removal efficiency was 60 min of precipitation, 14.5 mL of CaCl_2_ (3 mol/L), and calcination at 500 °C for 120 min. The synthesis condition that achieved the highest removal efficiency was 120 min of precipitation, 22 mL of CaCl_2_ (3 mol/L), and calcination at 400 °C for 120 min.

Based on the analysis of variance (ANOVA—Table S3), together with the Pareto chart (Figure S1), it was found that the factor with the greatest influence on phosphate removal efficiency was calcination temperature (C), followed by the volume of CaCl_2_ solution (B) and their respective quadratic interactions. In contrast, precipitation time (A) and calcination time (D), as well as their quadratic interactions (A^2^ and D^2^) and combinations with other factors (AB, AC, AD, BD, CD), were not statistically significant (*p*-value >0.05). Nevertheless, all first-order terms were retained in the polynomial equation for predicting the results. Only the interactions BD, AC, AD, and BC were removed. As a result, the adjusted coefficient of determination (*R*_adj_^2^) increased from 0.6726 to 0.7368. The exclusion of other factors, even when not significant, led to poorer model fitting, with reductions in coefficient of determination (*R* ^2^) and *R*_adj_^2^, which is why they were retained. The model equation in uncoded units is presented in eq S17.

Table S2 presents the experimental values alongside the predicted values obtained using the polynomial equation of the reduced model. Figure S2 illustrates the model’s ability to predict phosphate removal efficiency by BiMag.

In the analyzed response surfaces (Figure 1), the presence of curvature in all interactions highlights the significance of both interaction effects and quadratic terms, emphasizing the need to target intermediate factor values to maximize phosphate removal. It is observed that using the maximum values for calcination temperature (C) and calcination time (D) can compromise the material’s porous structure, promoting sintering, pore closure, and a consequent reduction in surface area, even though crystallinity and the formation of more stable CaFe_2_O_4_ phases increase. Similarly, excessive precipitation times (A) can lead to surface saturation of BiMag, reducing its available surface area, while high amounts of calcium (B) tend to block active sites and generate reagent waste. Conversely, very low values of precipitation time, CaCl_2_ volume, and calcination time result in lower incorporation of CaFe_2_O_4_ on the biochar surface, decreasing the availability of active sites and, consequently, the adsorbent’s efficiency.

**Figure 1:**
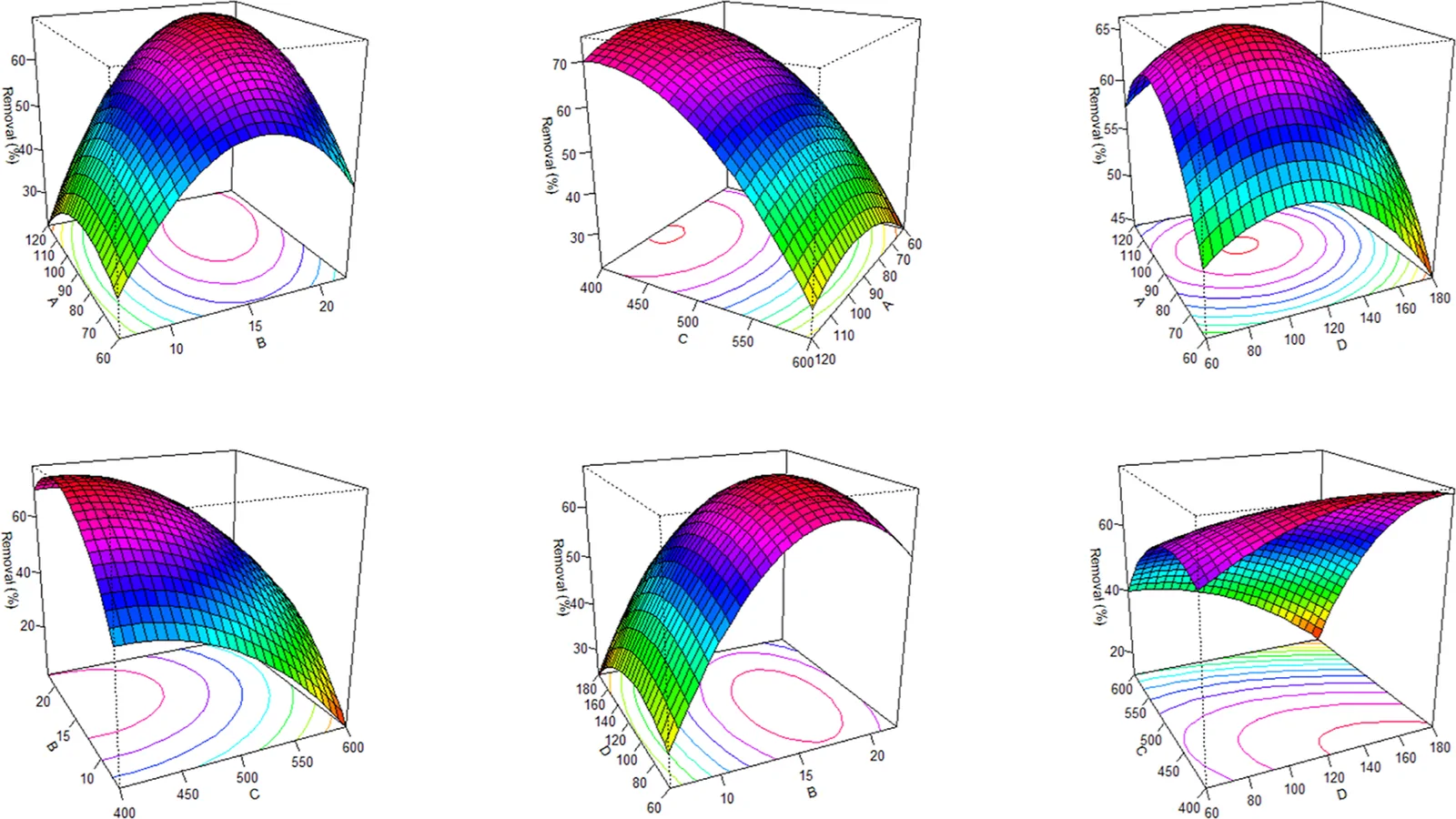
Response surfaces for optimization of phosphate adsorption on BiMag. Adsorption parameters: BiMag = 5 g/L; phosphate concentration = 10 mg/L; 150 rpm; 360 min; pH = 4; 20 °C.

Thus, restricted optimization was applied (Figure S3), which indicated the optimal conditions to maximize BiMag’s phosphate removal capacity as follows: 102 min of precipitation time (A), 18 mL of CaCl_2_ solution (B), and calcination at 400 °C (C) for 120 min (D). The selected precipitation time ensures proper structural formation, while the calcium amount promotes the generation of the CaFe_2_O_4_ phase on the biochar surface without causing excessive agglomeration. A temperature of 400 °C was sufficient to activate the Ca and Fe phases while remaining below the threshold for structural degradation, and the defined calcination time allows complete material transformation, preventing undesired sintering.

### 3.2 Effect of Precipitation Temperature

The effect of precipitation temperature on BiMag synthesis is shown in Figure 2.

**Figure 2:**
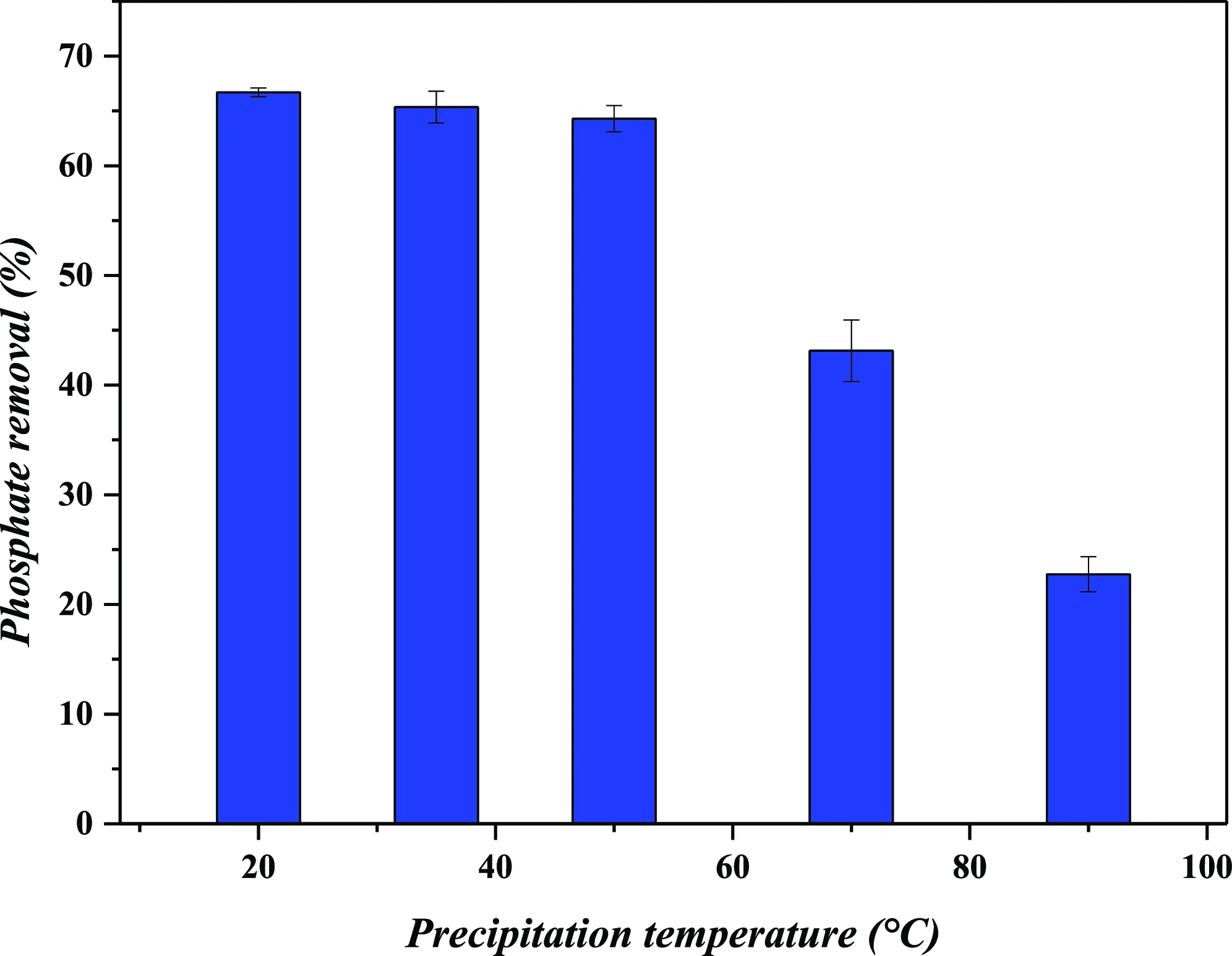
Temperature precipitation. Adsorption parameters: BiMag dosage = 5 g/L; phosphate concentration = 10 mg/L; pH = 4; contact time = 360 min; stirring = 150 rpm.

From Figure 2, it can be observed that increasing the precipitation temperature reduces the phosphate adsorption capacity of BiMag, with 20 °C showing the highest removal efficiency (66.70%), while 90 °C resulted in the lowest performance (22.75%). This behavior can be attributed to structural changes in the CaFe_2_O_4_ particles formed on the biochar surface, as lower temperatures favor the formation of smaller particles with a lower degree of crystallinity, higher porosity, greater density of surface active sites, and an amorphous character, features that enhance the availability of reactive sites for interaction with phosphate ions.

Precipitation at lower temperatures promotes abundant nucleation and slower particle growth, resulting in smaller structures with higher specific surface area and, consequently, a greater number of sites available for phosphate adsorption or complexation. Additionally, these conditions tend to preserve hydroxyl groups on the material’s surface, which can form stable complexes with phosphate ions through ligand exchange or oxygen bridging, thereby increasing the chemical affinity of BiMag. Similar results have been reported for the use of hematite in phosphate removal.^37^

Lower crystallinity, associated with a more amorphous character, promotes the formation of structural defects and disorders that act as additional adsorption sites. In contrast, higher precipitation temperatures favor particle growth and aggregation, reducing the surface area and inducing the crystallization of more stable but less reactive phases with fewer hydroxyl groups, thereby limiting the availability of active sites for interaction with phosphate.

Furthermore, the results obtained are directly related to the synthesis method employed. In the coprecipitation process, factors such as pH, reaction kinetics, agitation intensity, and diffusion rates of iron, calcium, and hydroxylated species play a crucial role in material formation. At lower temperatures, ionic mobility is limited, which restricts excessive growth of nuclei and favors the formation of smaller, more homogeneous, and well-dispersed particles on the biochar surface. Additionally, these conditions contribute to the preservation of hydroxylated or partially oxidized phases, known for their higher ion-exchange capacity with phosphate. Similar findings have been reported for the synthesis of magnetite nanoparticles.^38^

### 3.3 Effect of Iron Concentration

Figure 3 illustrates the effect of the volume of FeCl_3_ solution (3 mol/L) used in the preparation of BiMag on phosphate removal.

**Figure 3:**
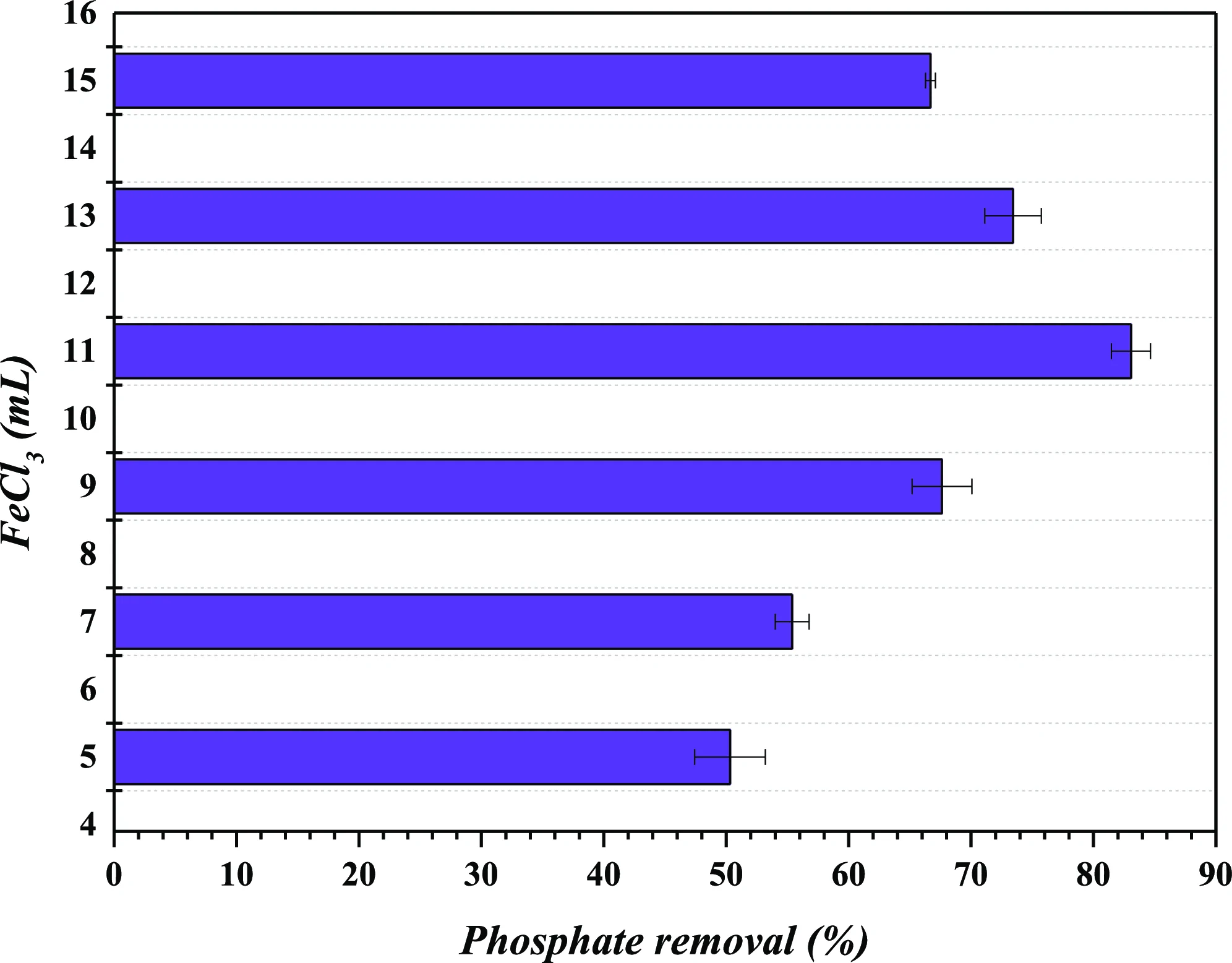
Iron effect. Adsorption parameters: BiMag dosage = 5 g/L; phosphate concentration = 10 mg/L; pH = 4; contact time = 360 min; stirring = 150 rpm; temperature = 25 °C.

As illustrated in Figure 3, phosphate removal efficiency progressively increased with higher Fe dosages until reaching an optimal value (11 mL of FeCl_3_—83% removal), after which a decline was observed. The increase up to the optimal concentration can be attributed to the fact that higher amounts of Fe during synthesis provide a greater number of active sites capable of interacting with phosphate species through complexation, precipitation, or ligand exchange. Furthermore, an appropriate Fe dosage promotes the formation of well-dispersed ferritic phases on the BiMag surface, resulting in a high surface area, adequate porosity, and availability of reactive functional groups. Previous studies with Fe­(III)-modified coconut shell biochar demonstrated that the presence of iron induces the formation of amorphous ferrihydrite, which enhances phosphate adsorption through surface ligand complexation, electrostatic interactions, and precipitation processes.^39^

Conversely, at Fe dosages above the optimal value, a decrease in phosphate removal efficiency is observed, likely due to several factors: aggregation of Fe nano- and microparticles, which reduces the dispersion and accessibility of active sites; partial blockage of BiMag porosity caused by excess precipitates or surface coverage, which decreases the surface area effectively available for adsorption; and saturation of active sites, meaning that additional Fe does not translate into greater interaction with phosphate species and may even lead to undesirable competitive effects.

In this context, a volume of 11 mL of FeCl_3_ (3 mol/L)—equivalent to an Fe concentration of 0.027 mol/L—suggests that this dosage allows an optimal balance between the availability of active sites, the dispersion of ferrite species, and the preservation of the adsorbent structure (biochar + CaFe_2_O_4_) with accessible surface area and open porosity.

This “bell-shaped” or “local maximum” trend has also been observed in other studies on biochar modification with Fe and Ca, where Fe content or Fe/Ca mineral loading beyond a certain point reduced effectiveness due to loss of accessibility and unfavorable morphological changes.^40^

Following this step, we concluded that the overall optimal condition to maximize the phosphate removal capacity of BiMag is as follows: 5 g of coffee grounds, 20 mL of H_2_O, 18 mL of CaCl_2_ (3 mol/L), 11 mL of FeCl_3_ (3 mol/L), 30 mL of NaOH (5 mol/L), 102 min of precipitation time at 20 °C, and calcination at 400 °C for 120 min. All subsequent adsorption experiments and characterizations were conducted using BiMag synthesized under these conditions.

### 3.4 Characterizations

FTIR was employed to investigate the functional groups present in the various materials prepared from coffee grounds and their modifications (Figure 4).

**Figure 4:**
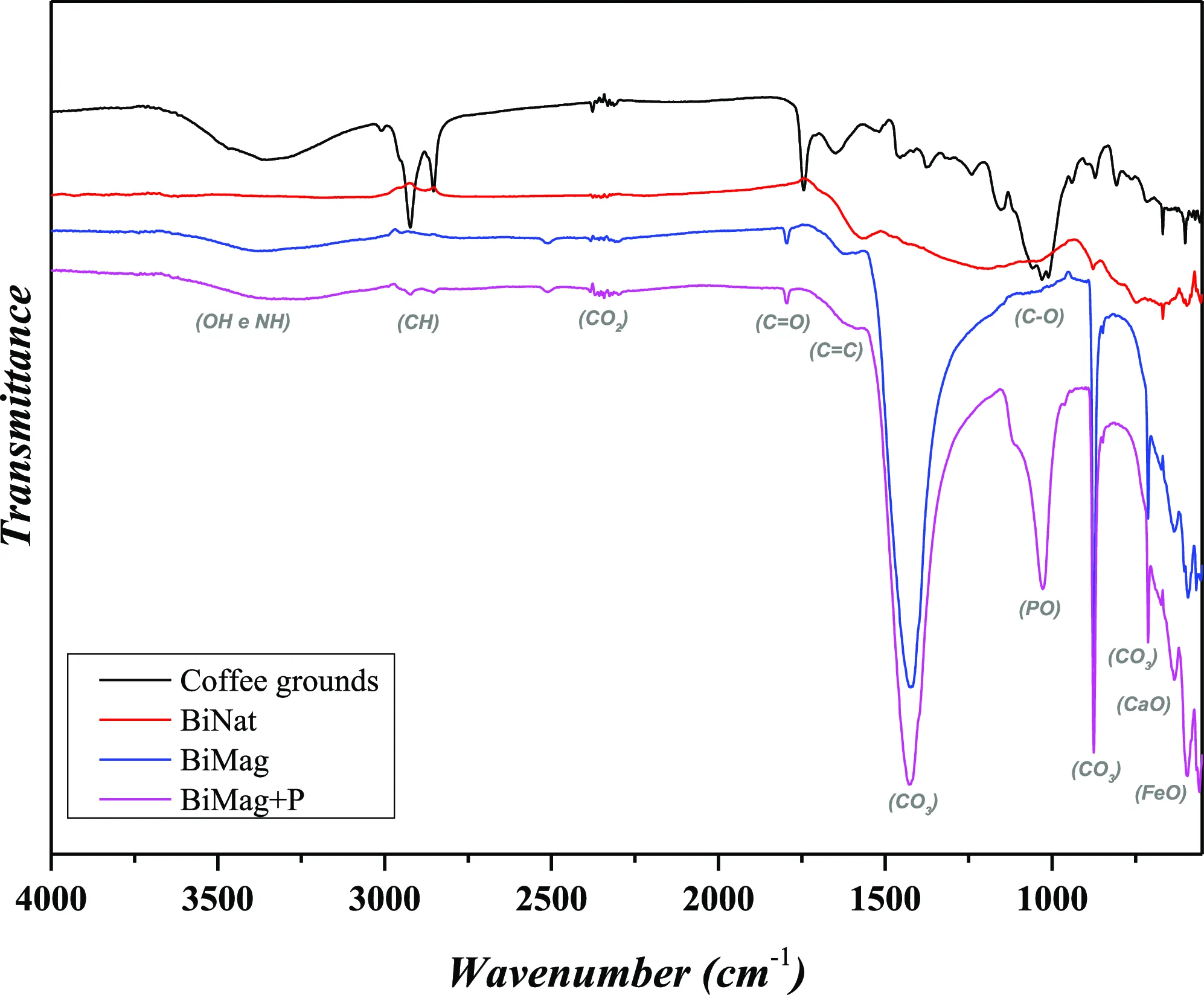
Surface functional groups of raw coffee grounds and biochars.

In the spectrum of raw coffee grounds, a broad band is observed around 3400 cm^–1^, attributed to the O–H stretching of alcohols, phenols, and carboxylic groups, as well as possible contributions from N–H groups originating from proteins. The bands at 2920–2850 cm^–1^ correspond to aliphatic C–H stretching, characteristic of cellulose, hemicellulose, and lipid chains. The peak at ∼1730 cm^–1^ is associated with C=O stretching of esters, aldehydes, and carboxylic acids, while the region at 1600–1510 cm^–1^ can be attributed both to aromatic C=C vibrations (lignin) and to N–H from proteins. Finally, the band at 1050–1030 cm^–1^ indicates the presence of C–O bonds from alcohols and polysaccharides. These results confirm that coffee grounds possess a complex lignocellulosic matrix, composed primarily of cellulose, hemicellulose, lignin, and nitrogenous compounds, as reported by Chen et al. (2008) and Tan et al. (2015).^41,42^

In the case of BiNat, the intensities of the bands at 3400 and 1730 cm^–1^ are significantly reduced, indicating the thermal degradation of cellulose, hemicellulose, and volatile oxygen-containing groups during pyrolysis. The persistence of a more pronounced band at ∼1600 cm^–1^ demonstrates an enrichment in aromatic structures, characteristic of carbonized materials. Furthermore, the reduced intensity in the 1050–1030 cm^–1^ region confirms the loss of oxygenated compounds, enhancing the material’s stability. These structural modifications are consistent with the literature, which reports increased aromaticity and recalcitrance of biochar after pyrolysis.^41,42^

The incorporation of CaFe_2_O_4_ into the biochar (BiMag) is confirmed by the appearance of a new band at 580–550 cm^–1^, attributed to Fe–O stretching vibrations. This band, absent in the unmodified biochar, verifies the successful magnetic impregnation. The spectrum also retains the characteristic features of BiNat, indicating that the modification did not completely eliminate the surface functional groups but added metallic active sites for interaction with ionic species. Similar results were reported by Mohammadi et al. (2025) when preparing magnetic biochars for phosphate removal.^43^

After the adsorption process, BiMag exhibited notable changes in the FTIR spectrum. The emergence and/or intensification of bands in the 1100–1000 cm^–1^ region, attributed to P–O stretching, confirms the immobilization of PO_4_^3–^ on the material’s surface. Additionally, the region around 1600 cm^–1^ showed intensity changes, suggesting possible interactions between the biochar’s aromatic groups and the phosphate species. The adjustment of the band at 580–550 cm^–1^ confirms the continued presence of the magnetic phase (Fe–O). The simultaneous presence of peaks at ∼1428 cm^–1^, 875 cm^–1^, and 712 cm^–1^ indicates CO_3_^2–^, consistent with the formation or retention of CaCO_3_ on the BiMag surface.^44^

The small peaks observed in the 2300–2337 cm^–1^ region in all spectra correspond to the characteristic absorption of atmospheric carbon dioxide (CO_2_), associated with the asymmetric stretching of the molecule.^45,46^ These signals are not related to the functional groups present in the analyzed materials but rather result from CO_2_ interference in the optical path of the equipment.

Therefore, the FTIR analysis highlights the following chemical transformations: (i) the coffee grounds exhibit functional groups characteristic of lignocellulosic biomass; (ii) pyrolysis produces a more aromatic and stable biochar, with a reduction in oxygen-containing groups; (iii) magnetization introduces FeO groups, indicating successful incorporation of CaFe_2_O_4_; and (iv) the adsorption of phosphate is confirmed by the emergence of characteristic PO bands in the BiMag spectrum after adsorption.

Raman spectroscopy of BiMag and BiMag + P revealed two well-defined maxima at approximately 1342 cm^–1^ and 1583 cm^–1^, corresponding to the D and G bands characteristic of carbonaceous materials, attributed respectively to structural defects (sp^3^ carbon/disorder) and the stretching of sp^2^-hybridized carbon atoms in graphitic structures (Figure 5). The D band at ∼1342 cm^–1^ indicates the presence of irregularities and defects in the carbon structure, possibly enhanced by the incorporation of CaFe_2_O_4_ particles into the carbon matrix, while the G band at ∼1583 cm^–1^ is associated with the graphitic network and sp^2^ order, suggesting that the biochar’s graphitic structure is preserved even after chemical modification. Comparing the spectra before and after phosphate adsorption, a slight increase in the D band intensity and a minor modification in the G peak shape were observed, which can be interpreted as a relative increase in surface defects and carbon–phosphate interactions on the material’s surface after adsorption. Similar results were reported by Joshi and Ragupathy and by Ali and colleagues when working with carbon-based adsorbents and a biochar–perovskite composite, respectively.^47,48^

**Figure 5:**
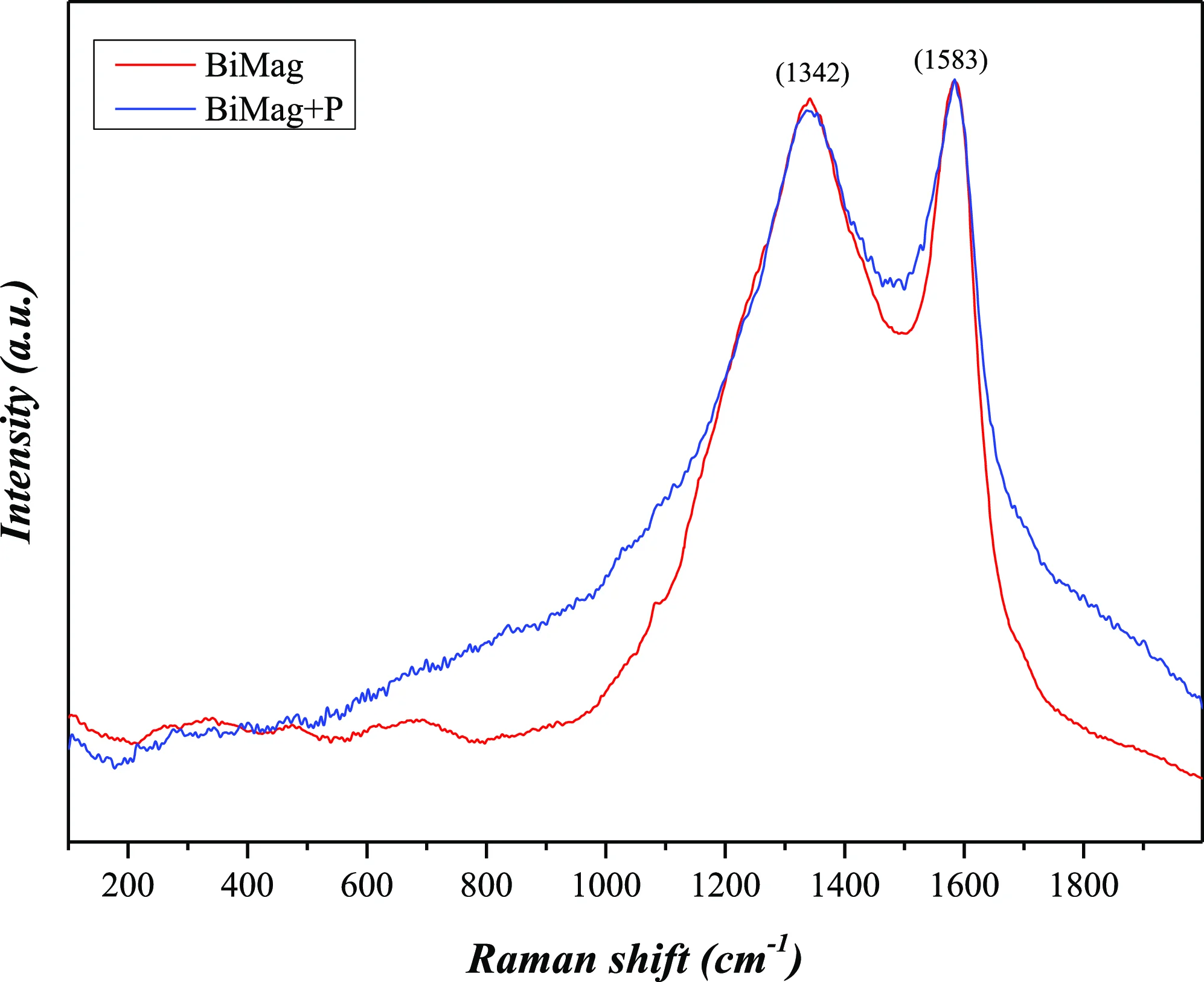
Raman spectroscopy of BiMag before and after adsorption.

Based on the SEM images, the morphological characterization of the BiNat and BiMag samples can be conducted (Figure 6).

**Figure 6:**
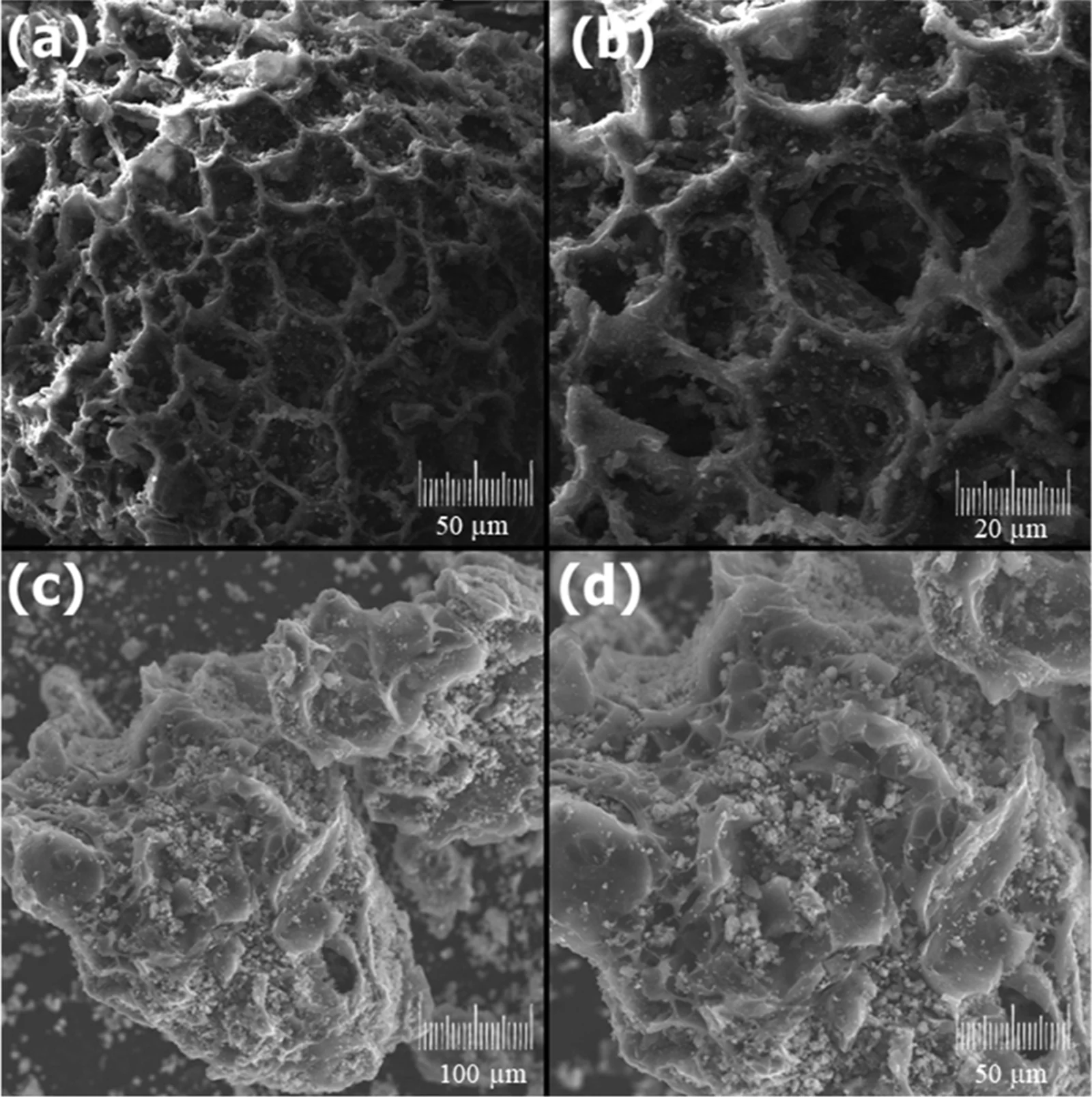
Morphological analysis of biochars. (a) BiNat, amplification 500×, (b) BiNat, amplification 1000×, (c) BiMag, amplification 450×. (d) BiMag, amplification 850×.

Figure 6a,b reveal the structure of BiNat. The observed morphology is porous and alveolar, featuring interconnected cells. The structure is characterized by a well-defined network of pores, resulting from the carbonization of the original organic matter. This porosity is crucial for the application of biochar, as it increases the surface area, which is essential for adsorption processes. The shape and arrangement of the pores resemble a honeycomb or spongy structure, typical of carbonaceous materials derived from plant biomass. This architecture reflects the cellular anatomy of coffee grounds, which was preserved after the pyrolysis process.

Figure 6c,d show the morphology of BiMag. The sample’s appearance is drastically different from that of the natural biochar. The sample now consists of aggregates of irregularly shaped particles of varying sizes. The presence of microparticles, either spherical or granular, dispersed across the surface and among the larger aggregates, is clearly visible. These particles are CaFe_2_O_4_, which agglomerated and adhered to the biochar structure during the functionalization process. The granular and aggregated morphology suggests that the pores of BiNat were covered by the CaFe_2_O_4_ particles.

The functionalization resulted in a significant morphological change, transforming the structure of BiNat into a denser architecture composed of aggregates of micro and nanoparticles. These particles, corresponding to the incorporated CaFe_2_O_4_, play a crucial role in the adsorption mechanism. Their deposition on the surface of BiMag is primarily responsible for phosphate removal, facilitating effective adsorption on the composite’s surface.

Although the SEM images indicate that the deposition of CaFe_2_O_4_ onto the biochar promotes partial pore filling or blockage, this does not necessarily imply a reduction in adsorption capacity.

Several studies have demonstrated that materials with low surface area or reduced porosity may still exhibit high phosphate adsorption performance when they possess chemically reactive surfaces. In this context, the presence of Ca and Fe species on the composite surface likely plays a more significant role than the complete preservation of the original porous structure of the biochar.^20,40^

Amorphous iron hydroxides, ferrihydrite, and goethite, for example, exhibit moderate surface areas but high affinity for phosphate due to the formation of highly stable inner-sphere complexes.^3,18,30^ In contrast, some highly porous materials, such as nonfunctionalized activated carbons, exhibit limited phosphate removal capacity precisely because of the absence of specific metallic sites capable of strongly interacting with phosphate anions.^25^

Ou et al. observed that Fe/Ca-modified biochars exhibited a significant reduction in surface area after metal modification but showed a substantial increase in phosphate removal due to the generation of new chemically active Fe–OH and Ca–OH surface sites.^20^ A similar phenomenon was reported by Yu et al., who observed that biochars rich in Ca and Fe minerals derived from paper mill sludge exhibited high phosphate adsorption despite their limited porous structure, attributing the performance primarily to calcium phosphate precipitation and surface complexation with ferric species.^40^

Thus, the high adsorption capacity observed for the biochar/CaFe_2_O_4_ composite suggests that the dominant mechanism is more strongly related to the chemical nature of the surface than exclusively to the preservation of the biochar porosity. Even with partial reduction of the porous structure, the incorporation of CaFe_2_O_4_ likely substantially increased the density of active sites capable of promoting chemical adsorption and surface precipitation of phosphate, thereby compensating for any loss of accessible surface area.

The chemical composition and elemental mapping of BiNat and BiMag, before and after phosphate adsorption, are illustrated in Figure S4. For BiNat, the dominant elements are C (76.27%), O (21.77%), Ca (1.35%), and K (0.61%). In BiMag, a greater variety of elements is observed due to modification with CaFe_2_O_4_. The dominant elements are Fe (30.99%), O (29.06%), Ca (21.59%), C (15.41%), and Cl (2.95%). After the adsorption experiments, P appears in the composition of BiMag, accounting for approximately 3.00%, and the O content also increases to 33.65%, confirming BiMag’s capacity to adsorb phosphate ions.

The structures of BiNat, BiMag, and BiMag + P are presented in Figure 7. BiNat exhibits the typical broad band characteristic of amorphous carbon, with prominent cellulose peaks (15 < 2θ < 30) that decrease in intensity in BiMag and BiMag + P, indicating partial degradation of the cellulosic and hemicellulosic components. Furthermore, these results suggest limited graphite growth, with the structure being predominantly composed of turbostratic carbon.^49^

**Figure 7:**
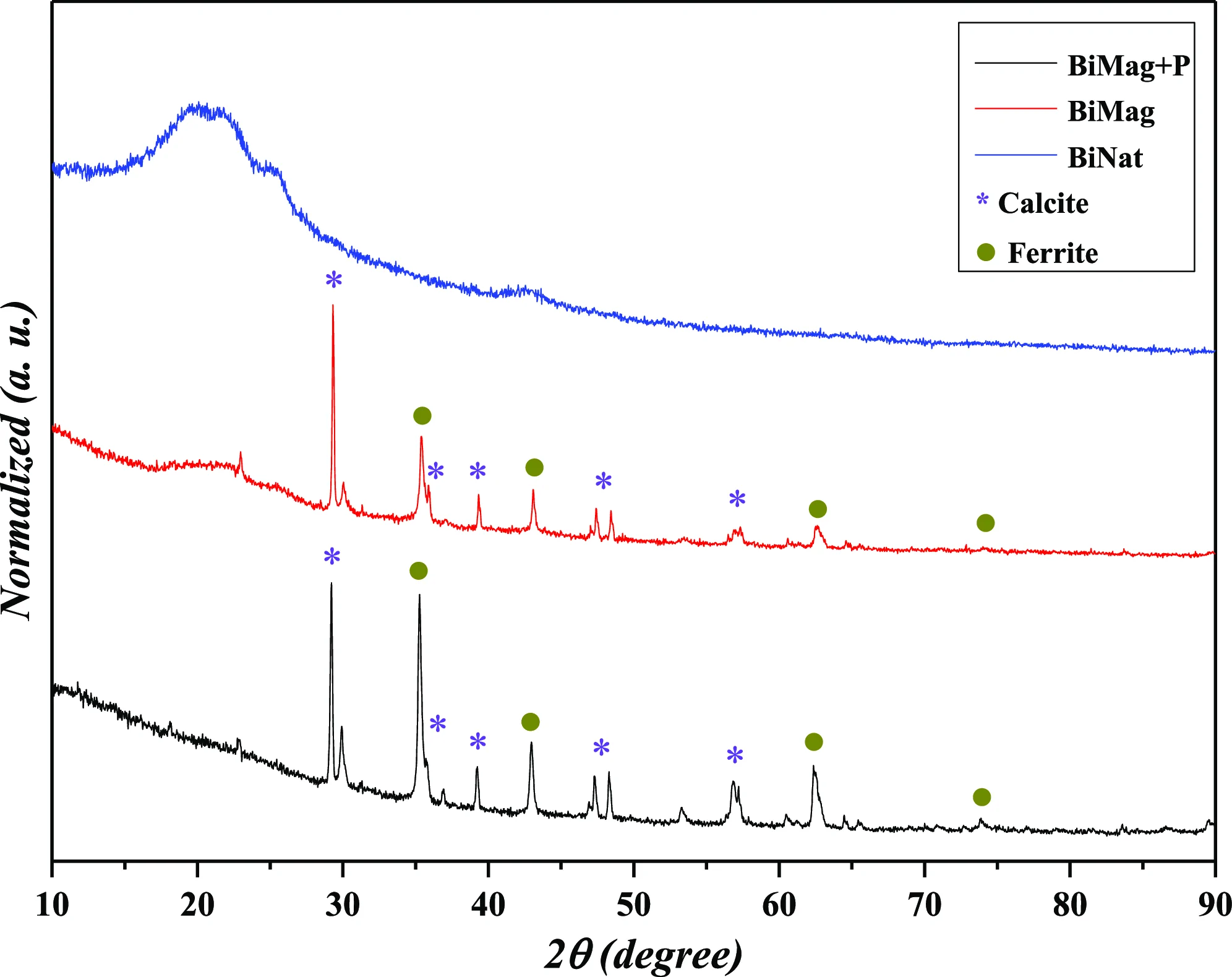
XRD analysis results.

BiMag exhibits crystalline peaks attributable to the CaFe_2_O_4_ and calcite (CaCO_3_) phases. The presence of these phases reduces the relative contribution of the amorphous hump and produces well-defined peaks. BiMag + P shows some peaks with greater intensity than those in BiMag, particularly those associated with calcium/oxide/carbonate phases.

The increased intensity of the crystalline peaks in BiMag + P may indicate the formation of additional crystals through the precipitation of calcium phosphate on the surface of BiMag (e.g., hydroxyapatite, brushite, or other Ca–P phases). These phases are crystalline and give rise to well-defined peaks in XRD. Consequently, the overall diffraction intensity at positions associated with Ca/Ca–P increases. Numerous studies on Ca-modified biochar have observed this exact behavior.^50^

The N_2_ adsorption–desorption isotherms for the sample BiMag is presented in Figure 8, accompanied by the BJH pore size distribution. The total surface area, calculated by BET method, is 87 m^2^/g, and the pore volume 0.15 cm^3^/g. The isotherm shape is consistent with type IV­(a), characteristic of mesoporous materials, and a discrete uptake in low pressure evidence the existence of a microporous system.^51^ The observed hysteresis possess features of both H3 and H4 types, indicating pores with a nonuniform size distribution. The profile of the desorption curve ending part, with an abrupt decrease of adsorbed gas, is typical of the cavitation phenomenon, which can be associated with partial blocking of the pores.^52^ Also, a narrow pore size distribution indicates homogeneity in cavities openings.

**Figure 8:**
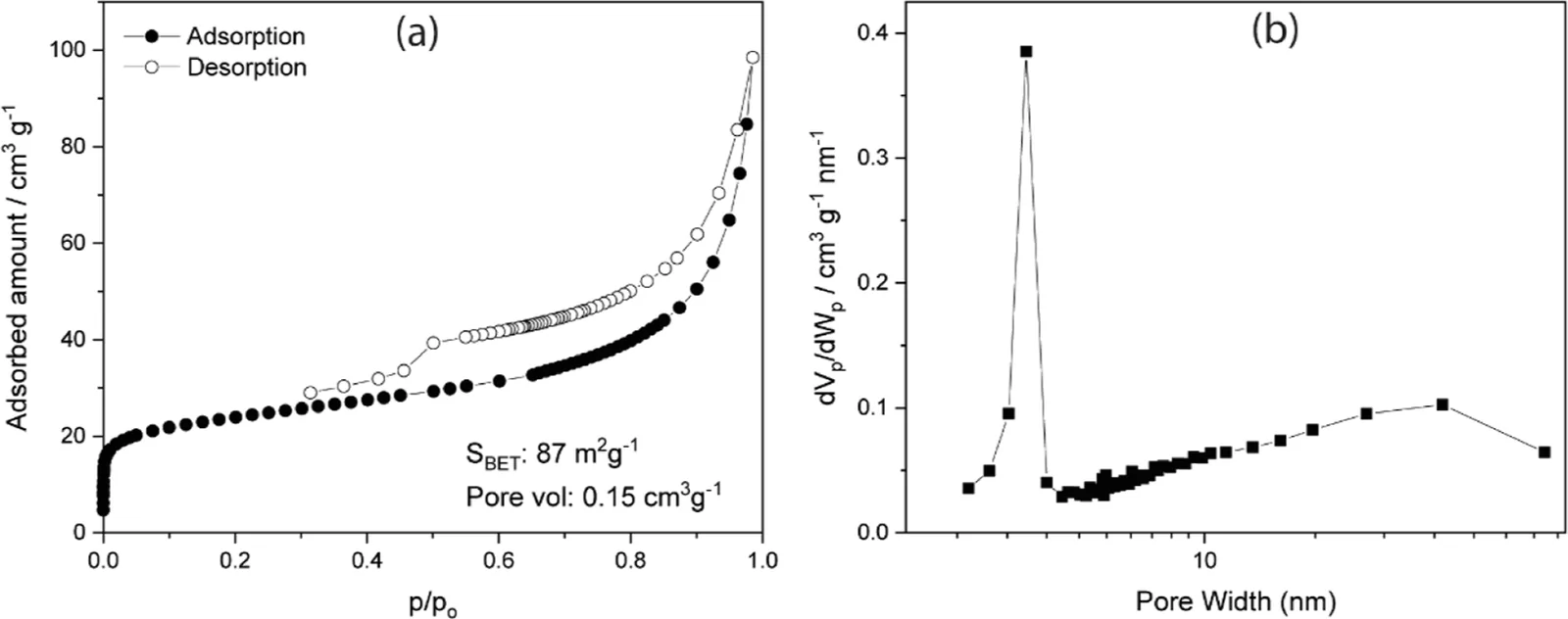
(a) N_2_ adsorption/desorption isotherms and (b) BHJ plots for the sample BiMag.

The magnetic response of BiMag was evaluated using VSM measurements over a magnetic field range of −14 to +14 kOe (Figure 9). The sample exhibited superparamagnetic behavior, with a saturation magnetization (Ms) of 22.50 emu/g. The remanent magnetization (Mr) and the squareness ratio (Mr/Ms) were 0.13 emu/g and 0.071, respectively. The superparamagnetic character is corroborated by the Mr/Ms value being below 0.1, indicating that BiMag can be magnetized under an external field, enabling faster and more efficient separation in aqueous solutions.

**Figure 9:**
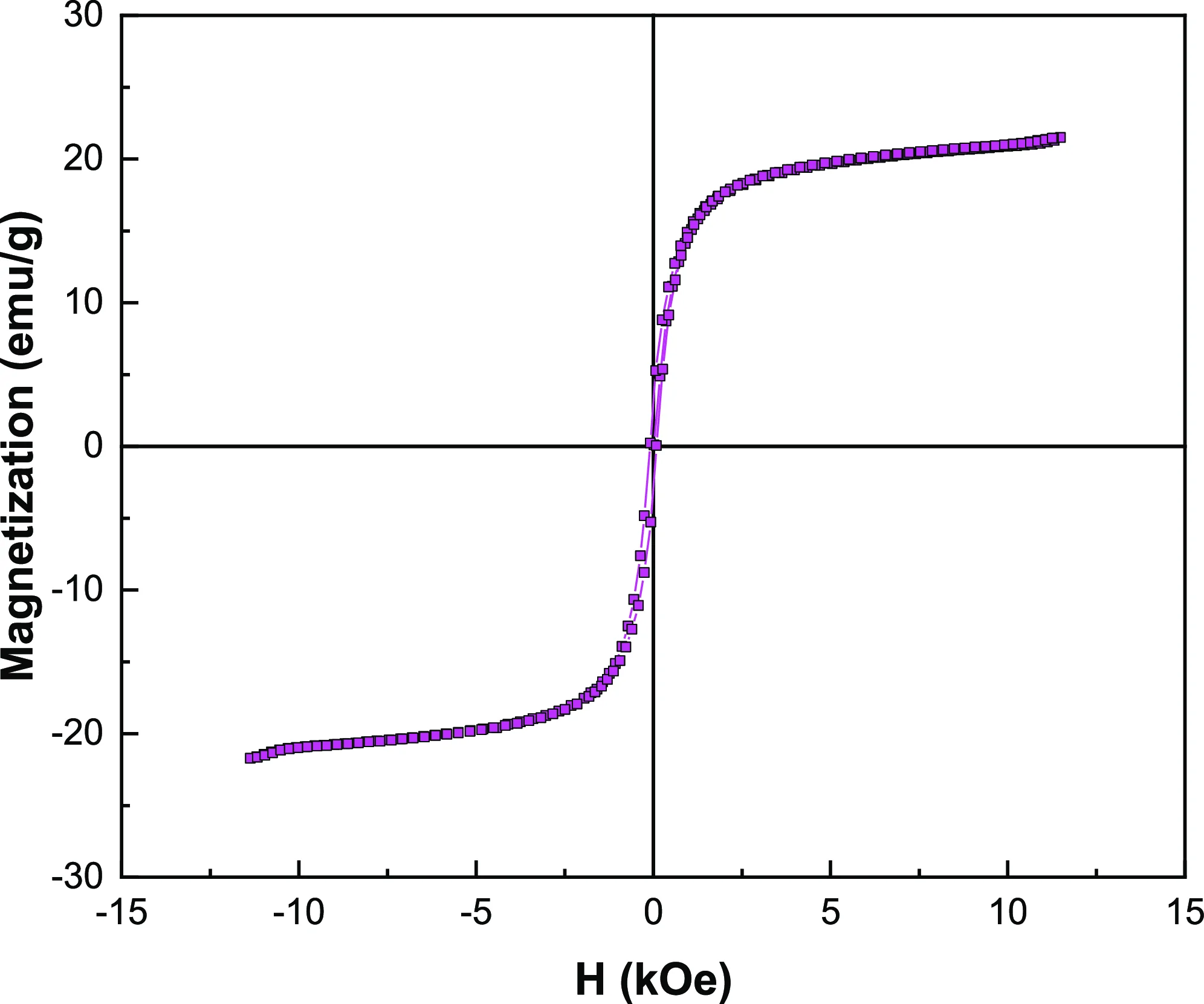
VSM magnetization curve of BiMag.

The pH at which BiMag exhibits no net surface charge was determined through the analysis of the pH_PZC_. As shown in Figure S5, the pH_PZC_ of BiMag is approximately 7.4. Therefore, to promote the interaction between BiMag and phosphate anions, the solution pH should be maintained below this value, ensuring that the BiMag surface is positively charged. Accordingly, all adsorption experiments were conducted at pH 4, a condition in which phosphate predominantly exists as dihydrogen phosphate (H_2_PO_4_^–^), which exhibits high affinity for BiMag.

### 3.5 Effect of Particle Size

The effect of BiMag particle size on phosphate adsorption is shown in Figure S6.

As shown in Figure S6, the smallest particle size (0.07 mm = 70 μm) resulted in the highest phosphate removal efficiency. This behavior is associated with an increase in the specific surface area available for adsorption, a reduction in intraparticle diffusion pathways, and improved contact between phosphate ions and the active sites of BiMag. In general, smaller particles expose more pores, reduce the thickness of the liquid film surrounding them, and decrease resistance to diffusion processes, thereby enhancing adsorption kinetics and, in some cases, increasing removal capacity. Nevertheless, the difference observed between the highest and lowest removal was only 6%, indicating that while particle size does exert an influence, the sizes evaluated do not vary sufficiently to significantly impact performance, likely because all of them are already within a relatively narrow range, in which further particle size reduction yields only marginal gains. Similar results have been reported for various adsorbent materials, such as granular ferric hydroxide, iron and aluminum oxi­(hydroxides), and coal residues, where decreasing particle size enhanced adsorption kinetics but the equilibrium removal efficiency remained similar across different size ranges.^53–55^

### 3.6 Effect of BiMag Dosage

Figure 10 illustrates the effect of BiMag dosage on phosphate removal at different initial concentrations (10, 30, and 50 mg/L). It can be observed that the removal efficiency progressively increases up to a dosage of 10 g/L, regardless of the concentration tested. This behavior can be attributed to the higher number of available active sites, the increase in effective specific surface area, and the improved dispersion of the adsorbent in the liquid phase, all of which favor interaction with phosphate ions. At higher phosphate concentrations, this increase is even more pronounced, as a greater amount of adsorbate is available to occupy the additional sites, resulting in a significant enhancement of the removal percentage until the optimal adsorbent-to-adsorbate ratio is reached.

**Figure 10:**
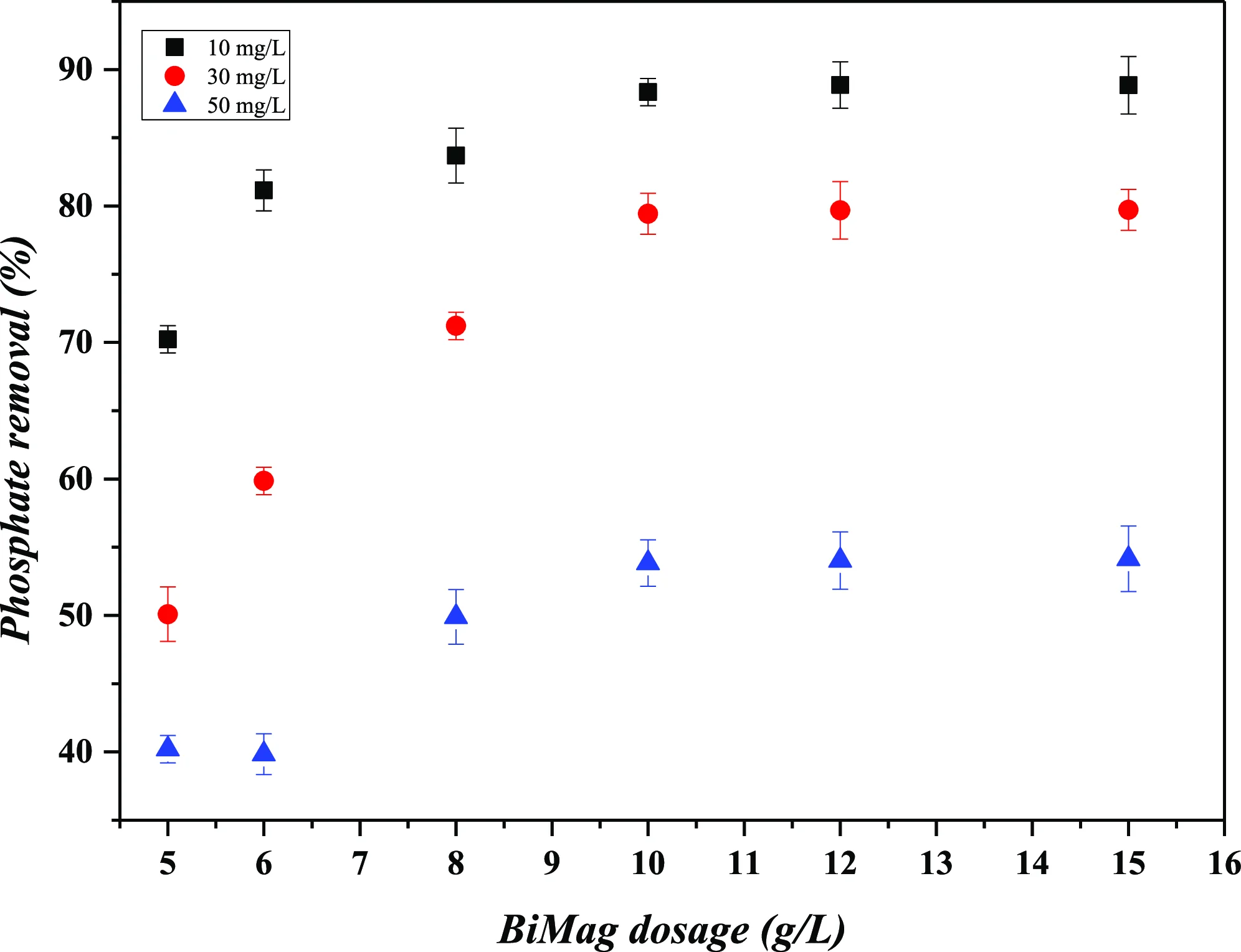
BiMag dosage. Adsorption parameters: pH = 4; contact time = 360 min; stirring = 150 rpm; temperature = 25 °C; BiMag granulometry = 70 μm.

When the BiMag dosage exceeds 10 g/L, the removal efficiency tends to plateau. This behavior can be attributed to two main factors: the aggregation of adsorbent particles, which reduces the effectively accessible surface area, and the saturation of active sites, since beyond a certain point, the most readily available sites are already occupied, making the process limited by slower diffusion mechanisms. Consequently, a dosage of 10 g/L was considered optimal and was used for all subsequent experiments.

This trend—an increase in efficiency up to an optimal dosage, followed by stabilization—is consistent with previously reported literature. Zhu et al. (2020), when evaluating a Fe–Mn–La composite adsorbent for phosphate removal, also observed that the removal efficiency increases with dosage until reaching a plateau, beyond which no significant additional gains are achieved.^56^

Although the optimal dosage obtained in this study (10 g/L) is relatively higher than that reported for some synthetic adsorbents with high surface areas, comparable values are frequently reported for modified biochars and waste-derived adsorbents applied to phosphate removal. The requirement for higher dosages in these materials is partially offset by the low cost of the precursors, ease of synthesis, and the potential for valorization of agro-industrial and industrial wastes.

Dalahmeh et al. evaluated iron- and calcium-impregnated biochars for decentralized wastewater treatment and observed relatively moderate adsorption capacities (1.5–6.4 mg/g), indicating that biochar-based systems often require larger adsorbent dosages to achieve high removal efficiencies in continuous applications.^57^

Zeng et al. reported that steel slag required approximately 40 g/L for efficient phosphate removal, whereas modified zeolitic clays required around 10 g/L. These results indicate that relatively high dosages are common among low-cost adsorbents intended for wastewater treatment.^58^

### 3.7 pH Effect

Figure 11 presents the effect of pH on phosphate removal. It can be observed that BiMag exhibited low sensitivity to pH variation, which represents a significant advantage compared to most iron-, aluminum-, or other metal-based adsorbents, which typically experience a marked decrease in efficiency under alkaline conditions due to surface deprotonation, increased anionic species, and the resulting electrostatic repulsion. In this context, BiMag’s ability to maintain high phosphate removal efficiency across a wide pH range underscores its potential for practical applications in the treatment of waters and effluents with variable characteristics.

**Figure 11:**
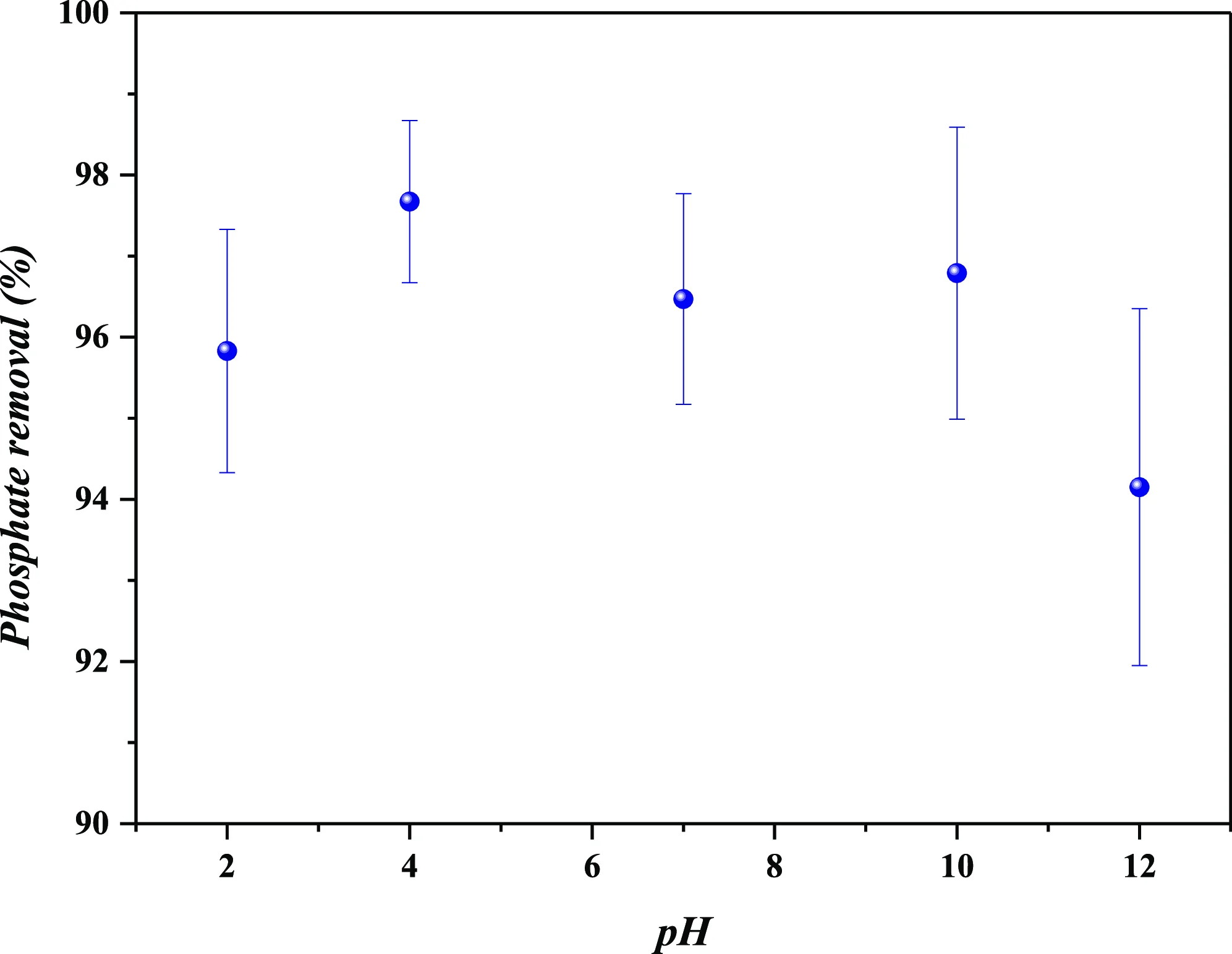
pH effect. Adsorption parameters: BiMag dosage = 10 g/L; phosphate concentration = 100 mg/L; contact time = 360 min; stirring = 150 rpm; temperature = 25 °C; BiMag granulometry = 70 μm.

The reason why pH has little effect on phosphate removal efficiency using BiMag is that multiple adsorption mechanisms act synergistically, so that even when changes occur in the adsorbent’s protonation state or in phosphate speciation, the material continues to provide effective active sites.

The presence of calcium promotes the formation of calcium phosphate phases, which exhibit relatively low solubility and, once formed, are less sensitive to moderate pH variations. Furthermore, the CaFe_2_O_4_ present on the material’s surface can provide ion-exchange or inner-sphere complexation sites, which perform effectively across a wide pH range. BiMag may also offer functional groups that are not strongly protonated or deprotonated; consequently, their interaction capacities are less dependent on pH.

Biochar/Ca–Fe composites tend to exhibit greater chemical resistance due to the carbonaceous matrix, which acts as a physical support and reduces metal leaching. This represents a crucial advantage, since many LDHs undergo partial dissolution or structural degradation under acidic or highly alkaline conditions.^59,60^

Similar results have been reported for phosphate adsorption using calcium-doped carbon and calcined dolomite powder.^61,62^

### 3.8 Coexistence of Anions

Figure 12 illustrates the effect of anion coexistence. It can be observed that the phosphate removal efficiency of BiMag hardly decreases with increasing concentrations of the anions (Cl^–^, NO_3_^–^, SO_4_^2–^, HCO_3_^–^, or CO_3_^2–^), indicating high selectivity for phosphate. This behavior suggests that the adsorbent’s active sites interact with phosphate through mechanisms that are stronger or more specific than simple electrostatic adsorption, resulting in minimal competition.

**Figure 12:**
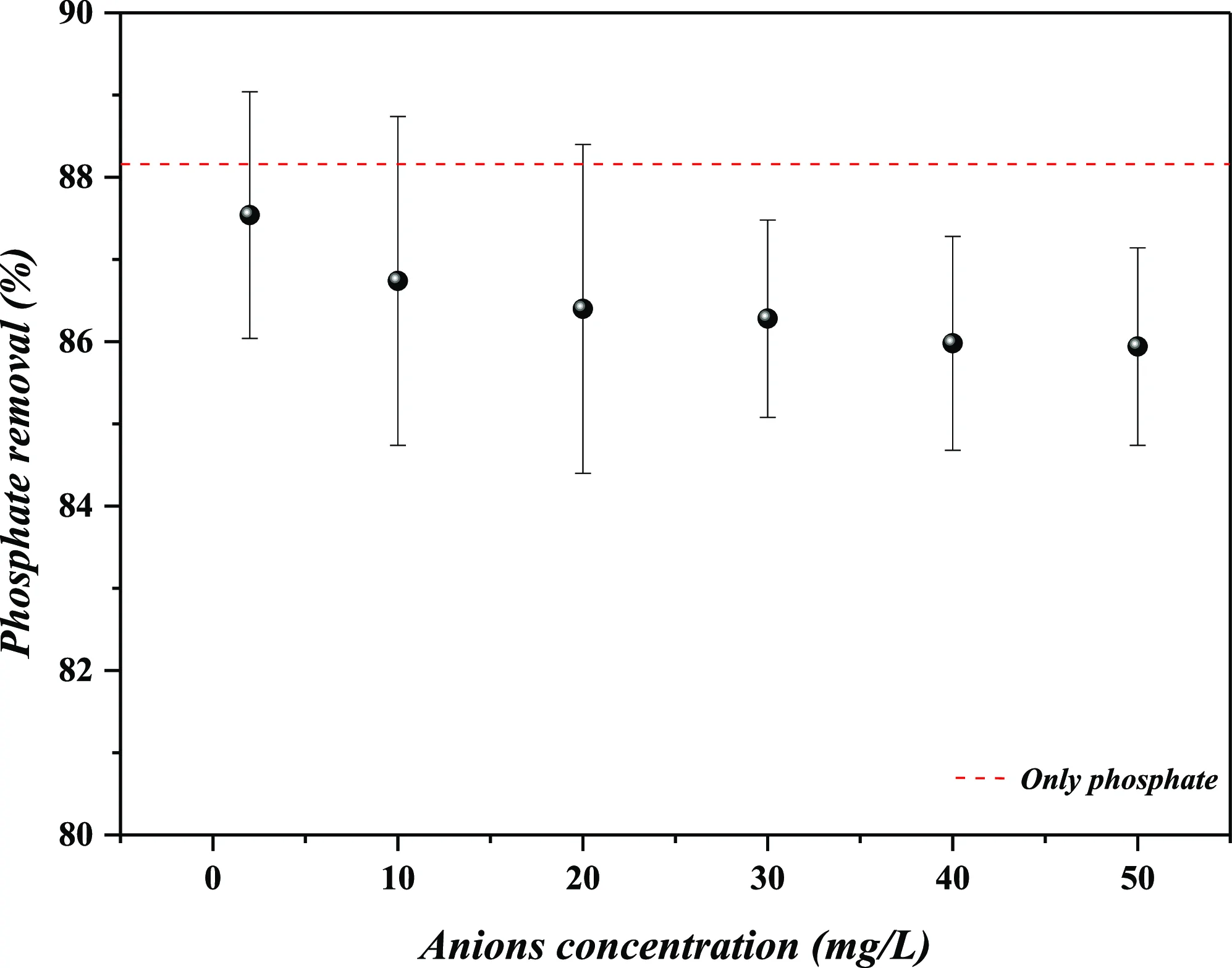
Coexisting anions. Adsorption parameters: BiMag dosage = 10 g/L; pH = 4; phosphate concentration = 10 mg/L; contact time = 360 min; stirring = 150 rpm; temperature = 25 °C; BiMag granulometry = 70 μm.

This result represents a significant advantage, as many adsorbents reported in the literature exhibit considerable decreases in efficiency in the presence of competing anions, particularly bicarbonate, carbonate, and sulfate, which can alter the pH, compete for active sites, or change the ionic strength.

Xu and collaborators found that the presence of anions significantly affects phosphate removal when using Mg–Fe layered double hydroxides. They further reported that the inhibition sequence follows the order: CO_3_^2–^ > SO_4_^2–^ > NO_3_^–^ > Cl^–^, with carbonate causing approximately 43% inhibition.^63^ Zeng and collaborators reported that anions such as CO_3_^2–^, HCO_3_^–^, NO_3_^–^, SO_4_^2–^, F^–^, and Cl^–^ affected the phosphate removal rate by less than 10% when using biochar modified with calcium and iron oxides, which is consistent with the observations in the present study and indicates high selectivity.^58^

Furthermore, Zhang and collaborators reported that compounds such as magnetite and goethite supported on biochar remove phosphate through ligand exchange, electrostatic attraction, and precipitation, and exhibit reasonable resistance to the presence of competing ions, depending on surface charge and pH.^64^

The simultaneous presence of calcium and iron generates multiple phosphate capture mechanisms. Iron acts primarily through surface complexation and ligand exchange between metallic hydroxyl groups and phosphate species, whereas calcium favors the precipitation of sparingly soluble calcium phosphates. This synergistic effect increases both the efficiency and selectivity of the adsorbent toward competing anions. In systems containing conventional LDHs, especially Mg/Al-LDH or Mg/Fe-LDH, phosphate removal frequently depends predominantly on anion exchange, making the material more susceptible to competition from carbonates and sulfates.^65^

### 3.9 Adsorption Kinetics

Figure 13 and Table S4 present the results obtained for contact time and adsorption kinetics modeling. As shown in Figure 13, the system reaches adsorption equilibrium after 240 min for a phosphate concentration of 10 mg/L, and after 300 min for phosphate concentrations of 50–100 mg/L.

**Figure 13:**
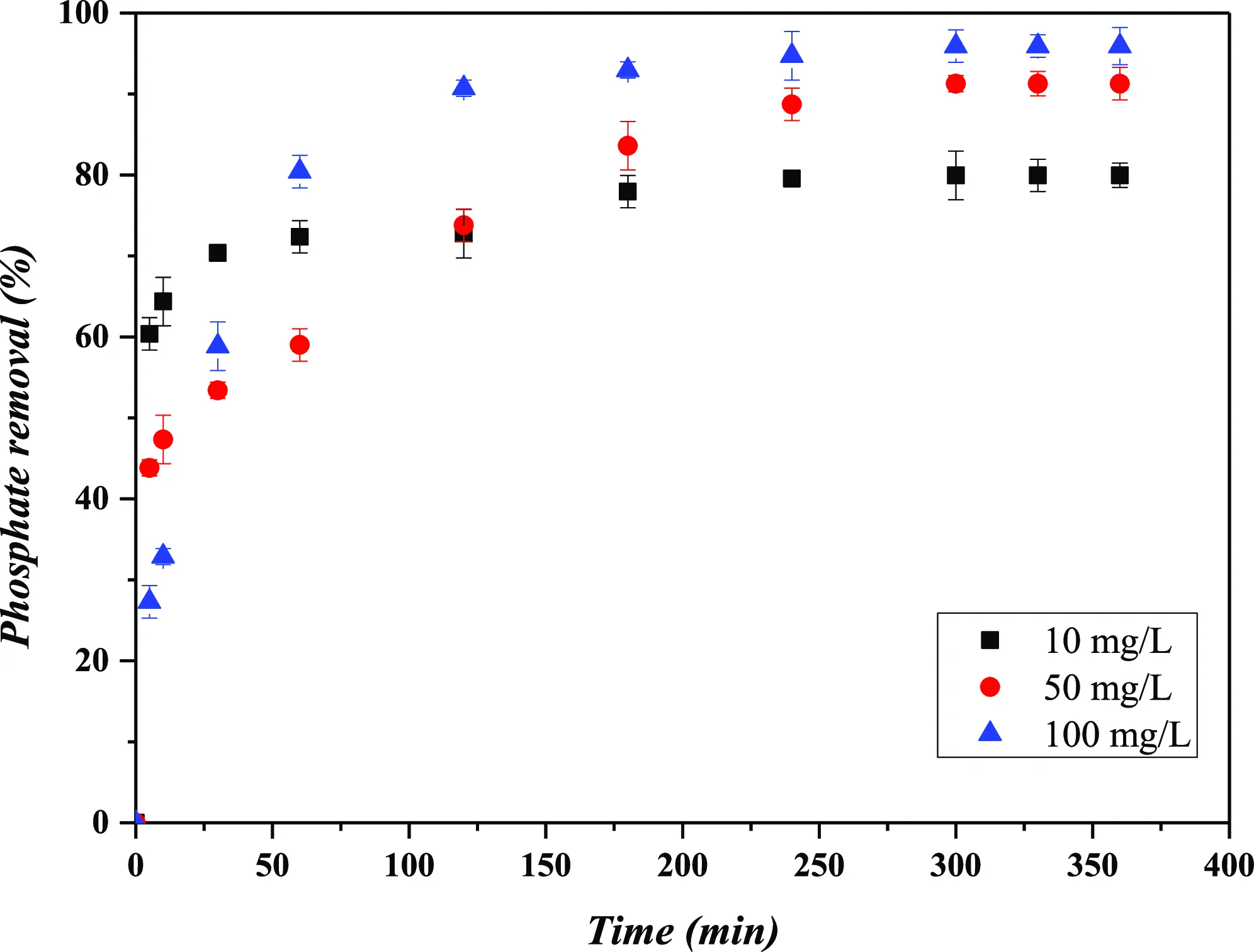
Contact time. Adsorption parameters: BiMag dosage = 10 g/L; pH = 4; stirring = 150 rpm; temperature = 25 °C; BiMag granulometry = 70 μm.

From Table S4, it can be observed that for initial phosphate concentrations of 10 and 50 mg/L, the Elovich model provided the best fit to the experimental data, exhibiting the highest adjusted *R* ^2^ values and the lowest RMSE values. This suggests that, in these cases, the adsorption mechanism is strongly influenced by adsorbent heterogeneities, activation/deactivation of surface sites, or a distribution of adsorption energies. For the initial phosphate concentration of 100 mg/L, the model that best fits the data is the PSO model, showing the highest adjusted *R*_adj_^2^ values and the lowest RMSE values.

The decrease in the Elovich model constants α and β with increasing initial phosphate concentration can be attributed to the availability of active sites on the adsorbent. At lower concentrations, the BiMag surface is less prone to saturation, which favors greater accessibility to reactive sites and, consequently, a higher initial adsorption rate (α). Similarly, the constant β, which represents the desorption rate over time, also tends to be higher under these conditions, reflecting the influence of processes such as partial surface saturation or diffusional limitations. As the phosphate concentration increases, competition among molecules for the available sites intensifies, leading to faster surface saturation. As a result, both α and β decrease, since site occupation occurs earlier and the kinetic process slows down over a shorter time interval.

This result has been observed in numerous studies and arises from several effects, such as (i) at higher concentrations, there is a greater density of adsorbate molecules in the solution, while the number of active sites on the adsorbent remains constant. Consequently, the available reactive sites are quickly exhausted, reducing the adsorption rate; (ii) higher concentrations can induce “blocking” or diffusion-limitation effects, where the denser molecules create transport barriers toward the pores or internal surfaces, decreasing the effective rate at which the adsorbate reaches all sites; (iii) saturation effects or repulsion between adsorbed molecules can also reduce the adsorption rate as the surface becomes increasingly occupied.

Similar results were reported by Orbuleţ and collaborators using Mg–Al–Fe layered double hydroxides, and by Missau and collaborators using CO_2_-activated biochar modified with Ca^2+^/Fe^3+^ for phosphate removal.^66,67^

### 3.10 Adsorption Isotherms

Figure 14 and Table S5 present the results obtained for the equilibrium study and adsorption isotherm modeling. As shown in Table S5, the Sips isotherm model provides the best representation of the data across the studied temperatures. Although at 25 °C the Langmuir model exhibits a slightly higher adjusted *R* ^2^ and a marginally lower RMSE, the fit of the Sips model is very close and, additionally, it predicts maximum phosphate adsorption capacities that are much closer to the experimental values, making this model more realistic for describing adsorption on the heterogeneous surface of BiMag.

**Figure 14:**
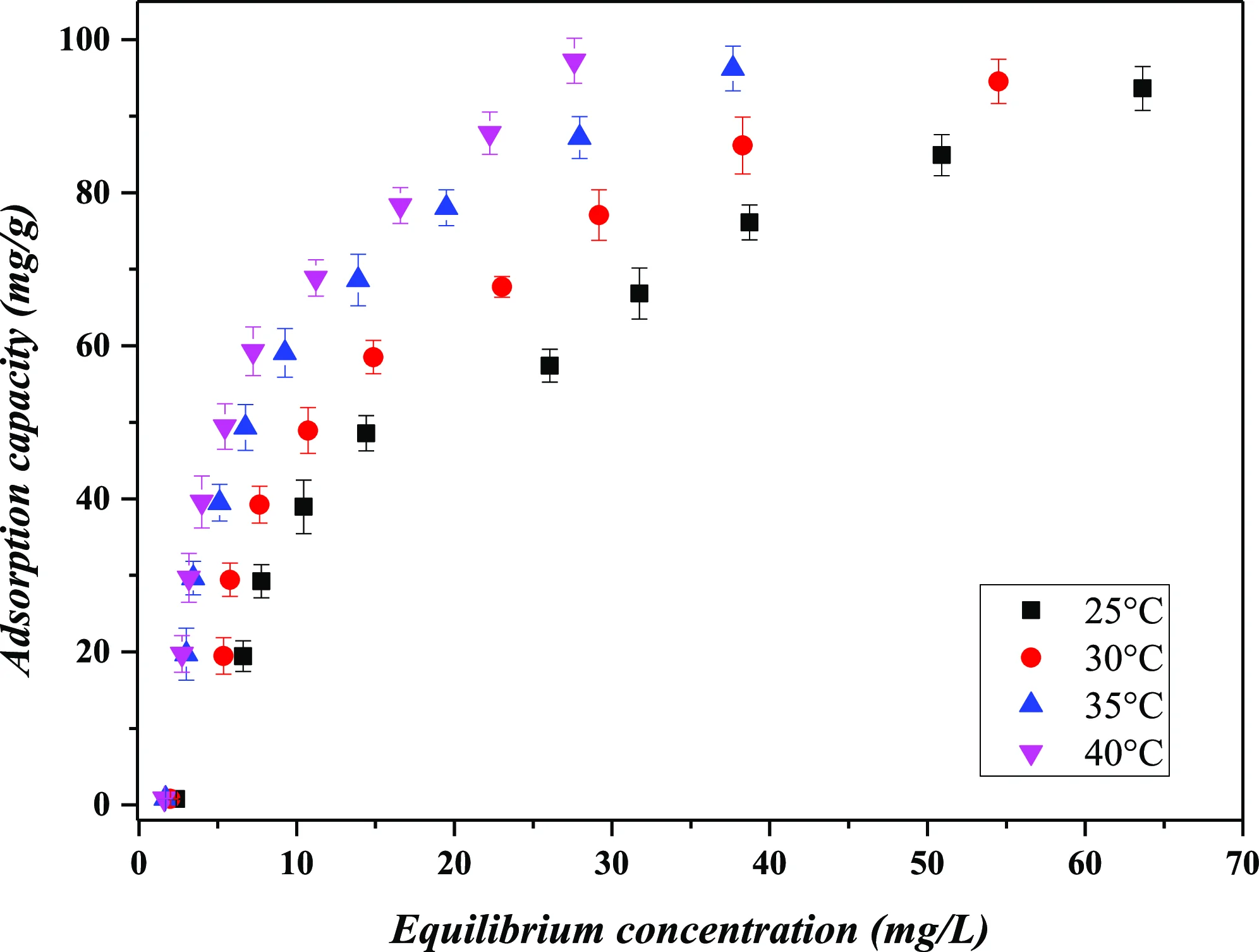
Equilibrium study. Adsorption parameters: BiMag dosage = 10 g/L; pH = 4; contact time = 15 h; stirring = 150 rpm; BiMag granulometry = 70 μm.

From Figure 14, it can be observed that increasing the temperature favors phosphate adsorption. This effect is evident from the steeper slope of the experimental curves, resulting from progressively lower equilibrium concentrations as the temperature rises. For example, the last experimental point on the 25 °C isotherm showed an equilibrium concentration and adsorption capacity of 63.64 mg/L and 93.64 mg/g, respectively. In contrast, the last point on the 40 °C isotherm—differing only in temperature from the corresponding point on the 25 °C isotherm—exhibited an equilibrium concentration and adsorption capacity of 27.61 mg/L and 97.24 mg/g, respectively.

The positive effect of increasing temperature on adsorption can also be observed in Table S5, where both the maximum adsorption capacities and the equilibrium constants increase with temperature. This suggests an endothermic process in which heating enhances the availability/reactivity of active sites (through increased mobility and/or greater solubility/interaction of phosphate ions with metal centers), a phenomenon previously reported for iron-modified biochars.^29,68,69^

The results from the D–R model show very high *E* values. Such elevated values indicate a strongly chemical nature of the phosphate adsorption process, consistent with complexation/binding at metal sites (Fe/Ca) and the formation of species strongly bound to the ferritic coating of BiMag. However, these values should be interpreted with caution, as they may reflect limitations of the D–R isotherm fitting. Therefore, strict interpretation of the numerical value of *E* should be approached prudently and always supported by complementary evidence.

### 3.11 Adsorption Thermodynamics

Table S6 presents the results obtained from the adsorption thermodynamics modeling. It can be observed that Δ*G*° is negative at all temperatures, indicating that the phosphate adsorption process is spontaneous under all these temperature conditions.

Furthermore, Δ*H*° is positive, showing that the process is endothermic and consistent with the experimental results from the isotherm study. The positive value of Δ*S*° also suggests an increase in disorder or greater randomness in the system during adsorption, possibly due to the release of water molecules when phosphate ions bind to the BiMag surface, or due to changes in the structure of the adsorbent/adsorbate interface.

Regarding the type of adsorption, the obtained Δ*G*° values (ranging from −46 to −50 kJ/mol) exhibit relatively high magnitudes. According to the literature, Δ*G*° values (in absolute terms) between 0 and 20 kJ/mol are typically attributed to physisorption, whereas values above 80 kJ/mol indicate chemisorption.^70,71^ In the present study, the Δ*H*° value of 49 kJ/mol also falls within an intermediate range, as chemisorption processes often exhibit enthalpies, in absolute terms, exceeding 80 kJ/mol. Therefore, the combined analysis of Δ*G*° and Δ*H*° suggests that the phosphate removal mechanism does not occur exclusively through chemisorption, but also involves contributions from physisorption and possibly ion-exchange processes.

The initial phosphate removal is predominantly governed by electrostatic attraction (physisorption) under conditions where pH < pH_PZC_. In this stage, negatively charged phosphate species (H_2_PO_4_^–^) interact with positively charged sites on the CaFe surface, particularly at lower pH values, where protonation of surface hydroxyl groups enhances electrostatic attraction. As the pH approaches or exceeds the pH_PZC_ value, chemisorption mechanisms become increasingly significant. Chemisorption is supported by the appearance of characteristic phosphate-related bands in the FTIR spectra, as well as by thermodynamic analyses indicating favorable adsorption behavior. In addition, ion-exchange mechanisms may contribute to phosphate uptake, in which H_2_PO_4_^–^ and HPO_4_^2–^ species replace OH^–^ or HCO_3_^–^ groups associated with the CaFe surface.^18^

### 3.12 Adsorption Mechanisms

The possible adsorption mechanisms involved are (i) inner-sphere complexation between Fe–OH or Ca–OH groups on BiMag and phosphate, resulting in stable covalent or coordination bonds that are minimally susceptible to displacement; (ii) chemical precipitation of calcium phosphate or iron/calcium phosphate on the BiMag surface; (iii) ligand exchange, in which OH^–^ groups or H_2_O molecules coordinated to the metal (Fe or Ca) are replaced by phosphate ions; (iv) electrostatic attraction, favored when BiMag exhibits a positive surface charge or sites with suitable polarization; and (v) the presence of the CaFe_2_O_4_ phase, which provides highly specific coordination sites for phosphate. The combined action of these mechanisms imparts high selectivity to the material, allowing phosphate to anchor stably to the BiMag surface. Figure 15 illustrates the mechanisms involved.

**Figure 15:**
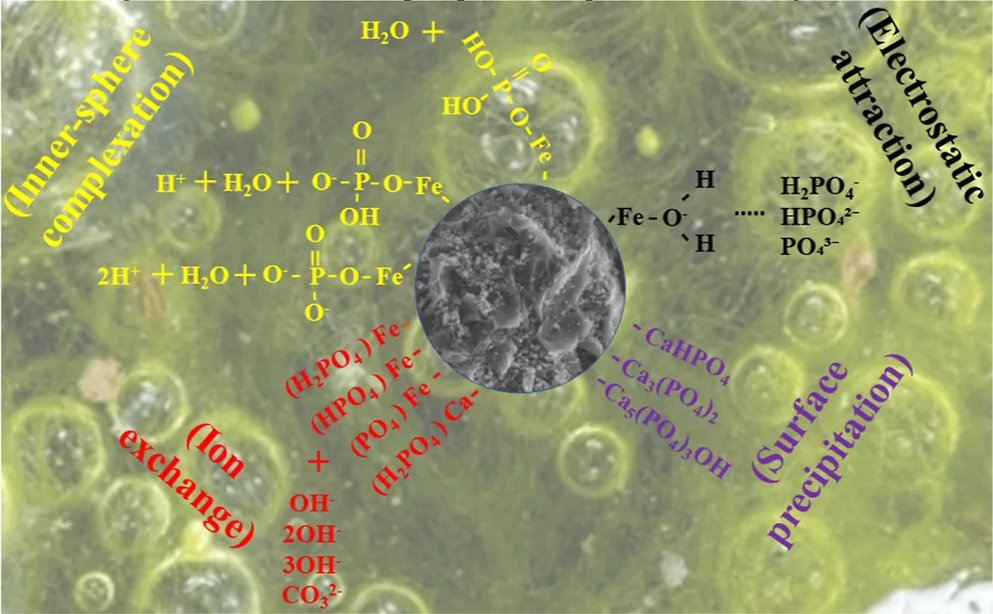
Mechanisms of phosphate adsorption on the BiMag surface.

### 3.13 Adsorption–Desorption Cycles

To evaluate the regeneration capacity of BiMag, adsorption–desorption cycle studies were conducted (Table S7). The results in Table S7 show that among the eluents tested, 0.1 mol/L NaOH best preserves the adsorption efficiency over successive regenerations. Eluents at lower concentrations tend to better maintain the adsorbent’s capacity throughout the cycles, whereas higher-concentration eluents can strongly compromise both adsorption capacity and regeneration performance.

Using 0.1 mol/L NaOH, the regeneration efficiency in the first cycle was 96.55%, whereas in the second, third, and fourth cycles the regeneration efficiencies were 92.16%, 63.95%, and 43.86%, respectively. At the end of the fourth adsorption cycle, the total amount of phosphate adsorbed was 2.23 mg/g. After the fourth desorption cycle, the amount of phosphate remaining impregnated on the BiMag surface was 0.44 mg/g, indicating that 1.79 mg of phosphate had been recovered. Therefore, the overall efficiency of the four adsorption/desorption cycles was 80.27%.

The decrease in adsorption and desorption efficiency over successive cycles can be attributed to several mechanisms. Among these are structural damage or changes in the physical properties of the adsorbent, leading to reduced surface area, pore blockage or collapse, and degradation of active groups. Additionally, the reduction in efficiency may be related to the formation of phosphate precipitates strongly bound to the BiMag surface.

Furthermore, it should be noted that after the adsorption cycles, BiMag becomes enriched with phosphorus due to phosphate retention within its structure, providing potential for direct application as a fertilizer or soil conditioner. This approach not only reduces costs and environmental impacts associated with continuous chemical regeneration but also promotes a closed nutrient cycle, in line with the principles of the circular economy.

Li and collaborators demonstrated that phosphate-enriched biochars offer advantages over conventional mineral fertilizers, particularly due to the slow and controlled release of phosphorus and the reduced quantities of the nutrient required to achieve high levels of phosphorus availability in the soil.^72^ Figueiredo and collaborators showed that biochars produced at 300–500 °C increase all soil phosphorus fractions, and that biochar produced at 300 °C maintained maize productivity comparable to mineral fertilization, indicating a residual effect and gradual release, highlighting its potential to reduce dependence on conventional phosphate fertilizers.^73^

Thus, the use of biochar after the adsorption process not only addresses the issue of the material’s limited regeneration but also adds value to the final product, transforming it into a useful source of phosphorus for agricultural soils.

## 4 Conclusions

The calcination temperature and the volume of CaCl_2_ are the most influential factors in the BiMag synthesis process. Characterizations confirmed the incorporation of the CaFe_2_O_4_ phase into the carbonaceous matrix, the formation of a superparamagnetic material, and the presence of active sites favorable for phosphate interaction. BiMag exhibited high removal efficiency, low pH sensitivity, and high selectivity against competing anions. Adsorption was well described by the Elovich and PSO kinetic models and the Sips isotherm model, indicating a heterogeneous surface. Thermodynamic analysis confirmed that the adsorption process is spontaneous and endothermic. Regeneration studies indicated that BiMag can be reused with moderate efficiency losses, and the phosphorus-enriched material shows potential for application as a fertilizer, contributing to nutrient recovery. Thus, BiMag emerges as a promising, low-cost, and renewable adsorbent with potential for wastewater treatment and sustainable phosphorus recovery.

## Supplementary Material



## Notes

Equations; adsorption experiments analyzed (Table S1); DOE for factor analysis (Table S2); ANOVA of factor analysis (Table S3); Pareto chart for analysis of significant effects (Figure S1); regression of the prediction model from the experimental results (Figure S2); restricted optimization for optimization of phosphate adsorption on BiMag (Figure S3); elemental composition of biochars (Figure S4); pH_PZC_ of BiMag (Figure S5); effect of particle size (Figure S6); results of adsorption kinetics modeling (Table S4); results of adsorption isotherms modeling (Table S5); adsorption thermodynamics parameters (Table S6); efficiency of adsorption–desorption cycles (Table S7) (PDF)
