# Supplementary material for: From Waste to Resource: Upcycling Spent Coffee Grounds into Biochar Functionalized with CaFe2O4 for Phosphate Removal
Source: ACS Omega. 2026 Sep 25;11(39):58996–9010. doi: 10.1021/acsomega.6c04188 (PMC13647842; doi:10.1021/acsomega.6c04188)
Supplement: Supplementary file 1 [file ao6c04188_si_001.pdf]

From Waste to Resource: Upcycling spent coffee grounds into biochar functionalized with $CaFe_2O_4$ for phosphate removal

Yago Neco Teixeira[a*], Marcella Araujo Macedo[b], André Vinicius Lopes Marques[b], Daniel Bernardes Silva[b], Jorge Marcell Coelho Menezes[b], Thiago Mielle Brito Ferreira Oliveira[b], Raimundo Nonato Pereira Teixeira[a], Adonay Rodrigues Loiola[c], Ronaldo Ferreira do Nascimento[d], Maria Gilnara Lima Bandeira[e], Mônica Belém Rodrigues[d], João Maria Soares[f], Meirielle Marques de Góis[f], Francisco José de Paula Filho[b]

[a]Biological Chemistry Department, Regional University of Cariri, R. Cel. Antonio Luis, 1161, Crato, CE 63105-000, Brazil.
[b]Science and Technology Center, Federal University of Cariri, Av. Ten. Raimundo Rocha, 1639, Juazeiro do Norte, CE 63048-080, Brazil.
[c]Department of Organic and Inorganic Chemistry, Federal University of Ceará, Fortaleza, CE 60440-900, Brazil.
[d]Analytical and Physical Chemistry Department, Federal University of Ceará, Fortaleza, CE 60356-000, Brazil.
[e]Materials characterization laboratory, Federal University of Ceará, Russas, CE 62900-000, Brazil.
[f]Physics Department, State University of Rio Grande do Norte, Mossoró, RN 59625-620, Brazil.
**Corresponding author: yago.neco@urca.br*

Equations

$$Y = \beta_0 + \sum_{i=1}^{n} \beta_i x_i + \sum_{i=1}^{n} \beta_{ii} x_i^2 + \sum_{i=1}^{n-1} \sum_{j=i+1}^{n} \beta_{ij} x_i x_j \quad (1)$$

$$D = \frac{0.9\lambda}{\beta Cos\theta} \quad (2)$$

$$q_t = \left(\frac{C_o - C_t}{m}\right)V \quad (3)$$

$$q_e = \left(\frac{C_o - C_e}{m}\right)V \quad (4)$$

$$Removal = \left(\frac{C_o - C_e}{C_o}\right)100 \quad (5)$$

$$q_t = q_e(1 - e^{-k_1 t}) \quad (6)$$

$$q_t = \frac{{q_e}^2 k_2 t}{1 + q_e k_2 t} \quad (7)$$

$$q_t = \frac{1}{\beta} \ln\,(1 + \alpha\beta t) \quad (8)$$

$$q_e = \frac{q_m k_L C_e}{1 + k_L C_e} \quad (9)$$

$$q_e = k_F {C_e}^{1/n} \quad (10)$$

$$q_e = \frac{q_m k_S {C_e}^n}{1 + k_S {C_e}^n} \quad (11)$$

$$q_e = q_m \exp\left\{ - k_{DR}\left[RT ln\left(1 + \frac{1}{C_e}\right)\right]^2 \right\} \quad (12)$$

$$E = \frac{1}{\sqrt{2 k_{DR}}} \quad (13)$$

$$\mathrm{k} = exp\left[\frac{\Delta S°}{R} - \left(\frac{\Delta H°}{R}\right)\frac{1}{T}\right] \quad (14)$$

$$\Delta G° = \Delta H° - T\,\Delta S° \quad (15)$$

$$Regeneration = \left(\frac{q_{des}}{q_e}\right)100\% \quad (16)$$

$$Y = -383 + 1.26A + 8.40B + 1.172C + 1.258D - 0.00899A^2 - 0.3302B^2 - 0.001173C^2 - 0.00182D^2 + 0.0324AB – 0.001747CD \quad (17)$$

Where:

x_i and **x_j** are the independent variables (precipitation time, $CaCl_2$ volume, calcination temperature, and calcination time);
Y is the dependent response variable (phosphate removal);
β_i, **β_{ii}**, and **β_{ij}** are the regression coefficients;
β_0 is the intercept coefficient;
D represents the average crystallite size perpendicular to the reflecting planes;
λ represents the X-ray wavelength;
β represents the full width at half maximum (FWHM);
θ is the diffraction angle;
q_t is the adsorption capacity at a given time (mg/g);
q_e is the adsorption capacity at equilibrium time (mg/g)
Removal is the removal efficiency of phosphate (%);
C_o is the initial concentration of phosphate (mg/L);
C_t is the concentration of phosphate at a given time (mg/L);
C_e is the concentration of phosphate at equilibrium time (mg/L);
m is the mass of BiMag used (g);
V is the volume of the solution (L);
k_1 and **k_2** are the adsorption rate constants for PFO (min^{-1}) and PSO ($g.mg^{-1}.min^{-1}$);
α is the initial adsorption rate ($mg.g^{-1}.min^{-1}$);
β is the desorption constant (mg/g);
t is the time (min);
k_L, **k_F**, **k_S**, and **k_{DR}** are the isotherm constants for the Langmuir (L/mg), Freundlich ($mg \cdot g^{-1} \cdot (mg \cdot L^{-1})^{-1/n}$), Sips (L/mg), and Dubinin–Radushkevich (mol^2/kJ^2) models, respectively;
n is the constant related to the heterogeneity of the adsorbent surface;
q_m is the maximum monolayer adsorption capacity (mg/g);
R is the universal gas constant (8.314 $J \cdot mol^{-1} \cdot K^{-1}$);
T is the process temperature (K);
E is the mean adsorption energy of the process (kJ/mol). Based on the value of E, the nature of the process can be assessed: physisorption ($E < 8$ kJ/mol), ion exchange ($8 < E < 16$ kJ/mol), or chemisorption ($E > 16$ kJ/mol);
ΔH° is the enthalpy change (kJ/mol);
ΔS° is the entropy change (kJ/mol);
ΔG° is the Gibbs free energy change (kJ/mol);
k is the equilibrium constant from the Langmuir isotherm (dimensionless). To render the equilibrium constant dimensionless, it was assumed that the activity coefficient of the dilute solution is equal to one;
q_{des} is the amount of phosphate desorbed (mg/g) after the contact between BiMag and the eluents;
Y is the phosphate removal (%);
A is the precipitation time (min);
B is the $CaCl_2$ volume (mL);
C is the precipitation temperature;
D is the precipitation time (min).

Table S1: Adsorption experiments analyzed.

Test	BiMag dosage (g/L)	Phosphate concentration (mg/L)	Coexisting anions* (mg/L)	Time (min)	Granulometry (mm)	Temperature (°C)	pH
Granulometry	5	10	0	360	0.07, 0.15, 0.25, 0.4	25	4
Adsorbent dosage	5, 6, 8, 10, 12, 15	10, 30, 50	0	360	0.07	25	4
pH	10	100	0	360	0.07	25	2, 4, 7, 10, 12
Isotherms and Termodynamics Adsorption	10	10, 200, 300, 400, 500,600, 700, 800, 900, 1000	0	900	0.07	25, 30, 35, 40	4
Kinetics Adsorption	10	10, 50, 100	0	0, 5, 10, 30, 60, 120, 180, 240, 300, 330, 360	0.07	25	4
Coexisting anions	10	10	2, 10, 20, 30, 40, 50	360	0.07	25	4

*Anions: Cl^-, NO_3^-, SO_4^{2-}, HCO_3^- and CO_3^{2-}.

Table S2: DOE for factor analysis.

Run	Real values				Response	
Order	A (min)	B (mL)	C (°C)	D (min)	Phosphate removal (%)	Predict (%)
1	60	7	500	120	23.95	29.15
2	120	7	500	120	23.15	21.26
3	60	22	500	120	43.92	40.67
4	120	22	500	120	72.28	61.95
5	90	14.5	400	60	63.89	60.36
6	90	14.5	600	60	49.52	39.19
7	90	14.5	400	180	69.89	75.05
8	90	14.5	600	180	13.57	11.95
9	60	14.5	500	60	51.11	50.07
10	120	14.5	500	60	61.50	56.76
11	60	14.5	500	180	48.72	43.79
12	120	14.5	500	180	51.11	50.49
13	90	7	400	120	59.50	42.63
14	90	22	400	120	49.92	68.73
15	90	7	600	120	15.17	0.50
16	90	22	600	120	25.15	26.60
17	60	14.5	400	120	71.48	62.82
18	120	14.5	400	120	67.89	69.51
19	60	14.5	600	120	11.17	20.69
20	120	14.5	600	120	15.17	27.38
21	90	7	500	60	14.37	29.88
22	90	22	500	60	55.11	55.98
23	90	7	500	180	14.37	23.61
24	90	22	500	180	60.70	49.71
25	90	14.5	500	120	61.50	64.92
26	90	14.5	500	120	65.09	64.92
27	90	14.5	500	120	69.89	64.92

*Adsorption parameters: BiMag = 5 g/L; Phosphate concentration = 10 mg/L; 150 rpm; 360 min; pH = 4; 20 °C.

Table S3: ANOVA of factor analysis.

Source	DF	Adj SS	Adj MS	F-Value	P-Value
Model	14	10443.70	745.98	4.82	0.005
Linear	4	7623.30	1905.81	12.30	0
A	1	138.30	138.30	0.89	0.363
B	1	2042.90	2042.92	13.19	0.003
C	1	5327	5327.04	34.39	0
D	1	115	115.01	0.74	0.406
Square	4	2034.20	508.55	3.28	0.049
A^2	1	348.90	348.89	2.25	0.159
B^2	1	1839.60	1839.64	11.88	0.005
C^2	1	734.10	734.12	4.74	0.05
D^2	1	228.10	228.13	1.47	0.248
2-Way Interaction	6	786.20	131.03	0.85	0.559
AB	1	212.50	212.54	1.37	0.264
AC	1	14.40	14.40	0.09	0.766
AD	1	16	15.97	0.10	0.754
BC	1	95.80	95.76	0.62	0.447
BD	1	7.80	7.82	0.05	0.826
CD	1	439.70	439.72	2.84	0.118
Error	12	1859	154.91		
Lack-of-Fit	10	1823.60	182.36	10.30	0.092
Pure Error	2	35.40	17.71		
Total	26	12302.60			

$R^2 = 0.8489$. $R_{adj}^2 = 0.6726$.

Figure S1: Pareto chart for analysis of significant effects.

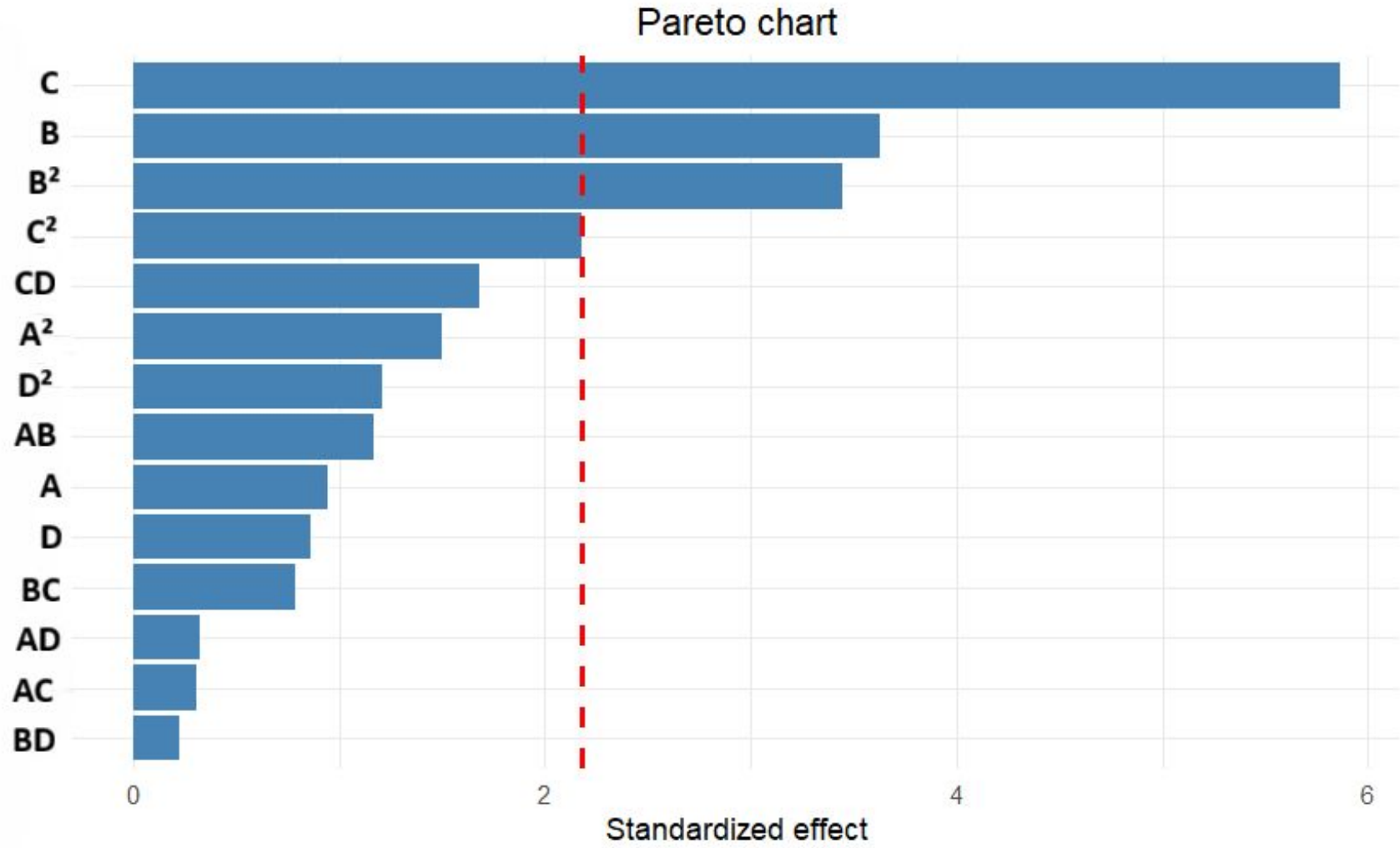

Figure S2: Regression of the prediction model from the experimental results.

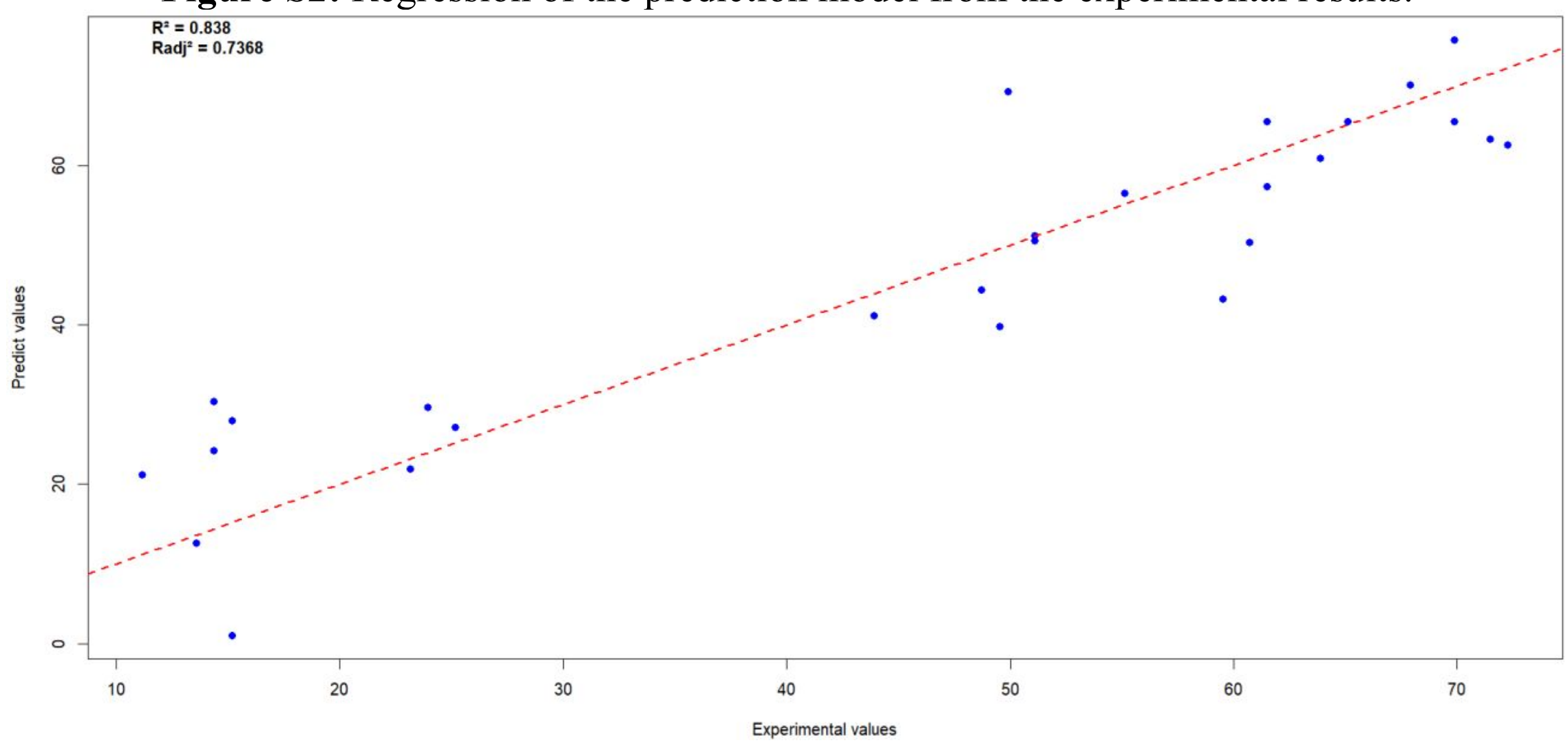

Figure S3: Restricted optimization for optimization of phosphate adsorption on BiMag. Adsorption parameters: BiMag = 5 g/L; phosphate concentration = 10 mg/L; 150 rpm; 360 min; pH = 4; 20 °C.

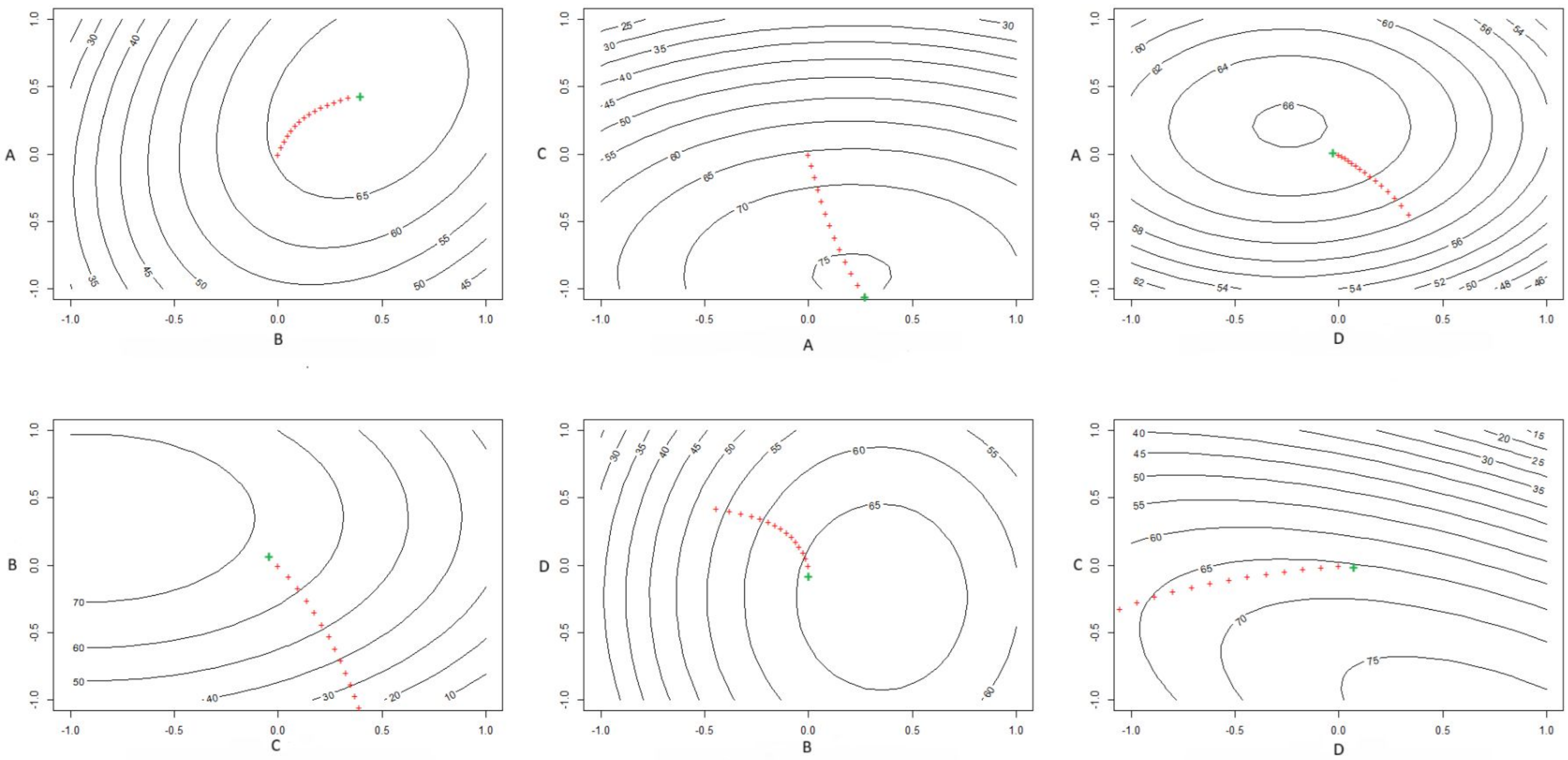

Figure S4: Elemental composition of biochars. (a) BiNat. (b) BiMag before adsorption of phosphate. (c) BiMag after adsorption of phosphate.

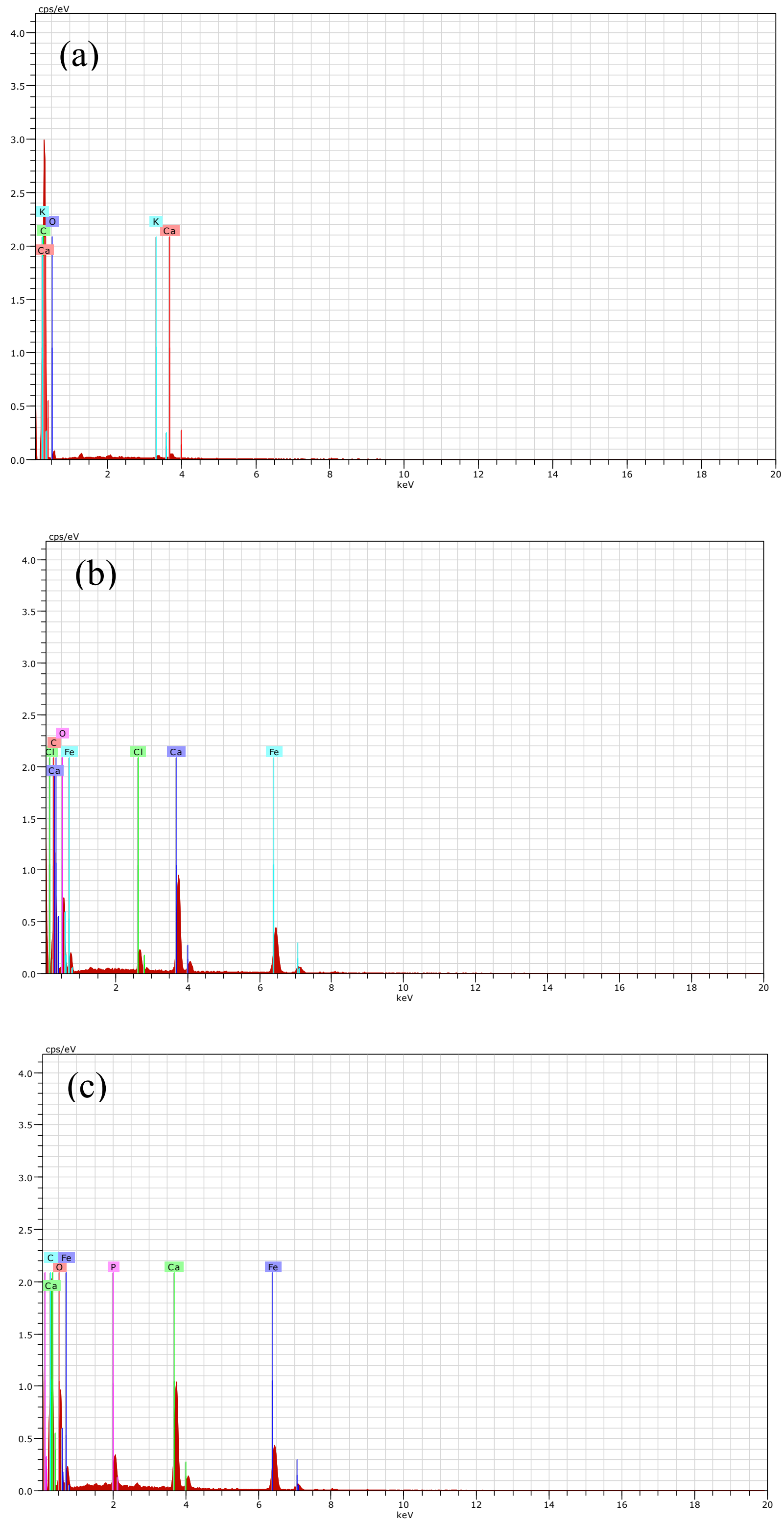

Figure S5: pH_{PZC} of BiMag.

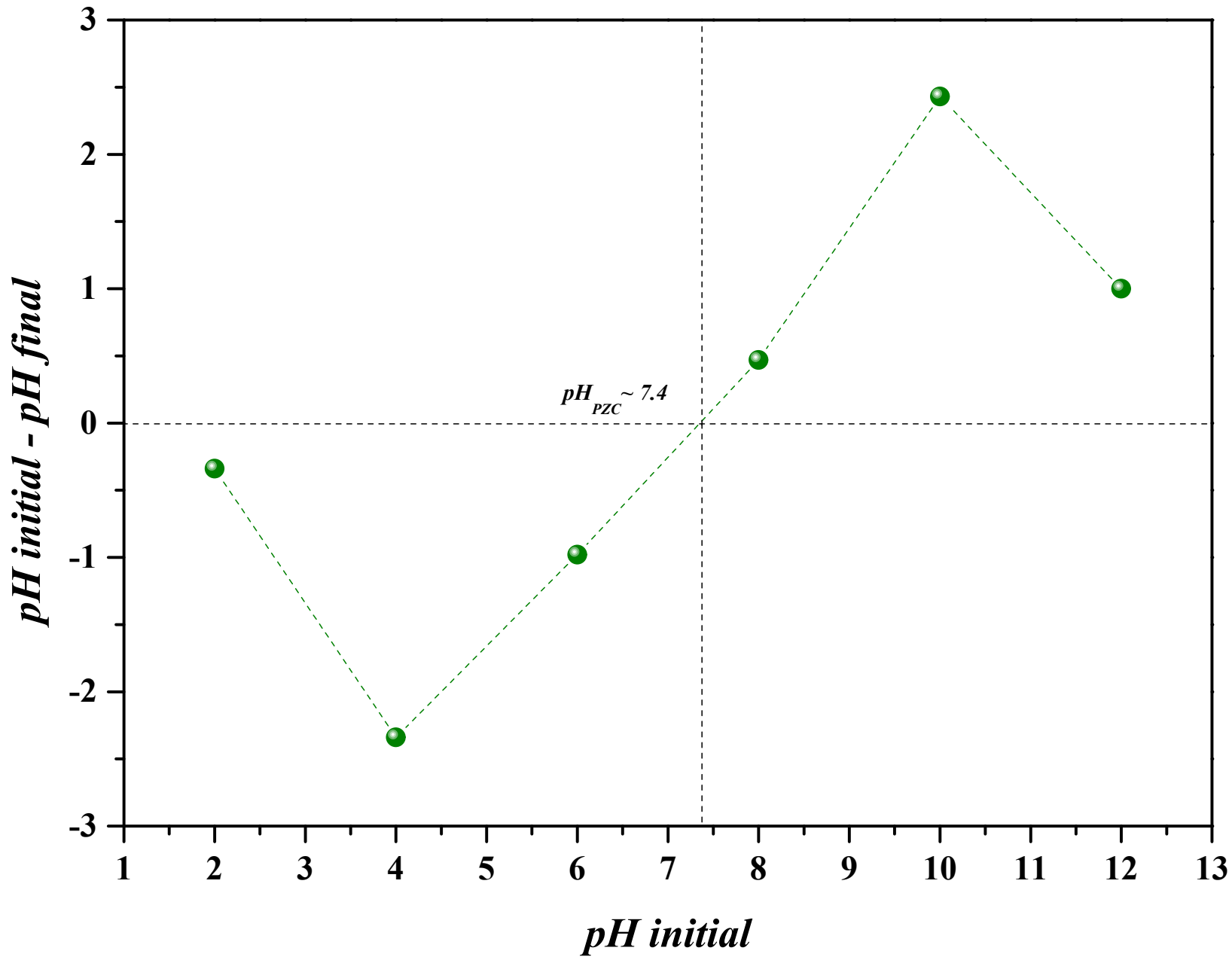

Figure S6: Effect of Particle Size. Adsorption parameters: BiMag dosage = 5 g/L; phosphate concentration = 10 mg/L; pH = 4; contact time = 360 min; stirring = 150 rpm; temperature = 25°C.

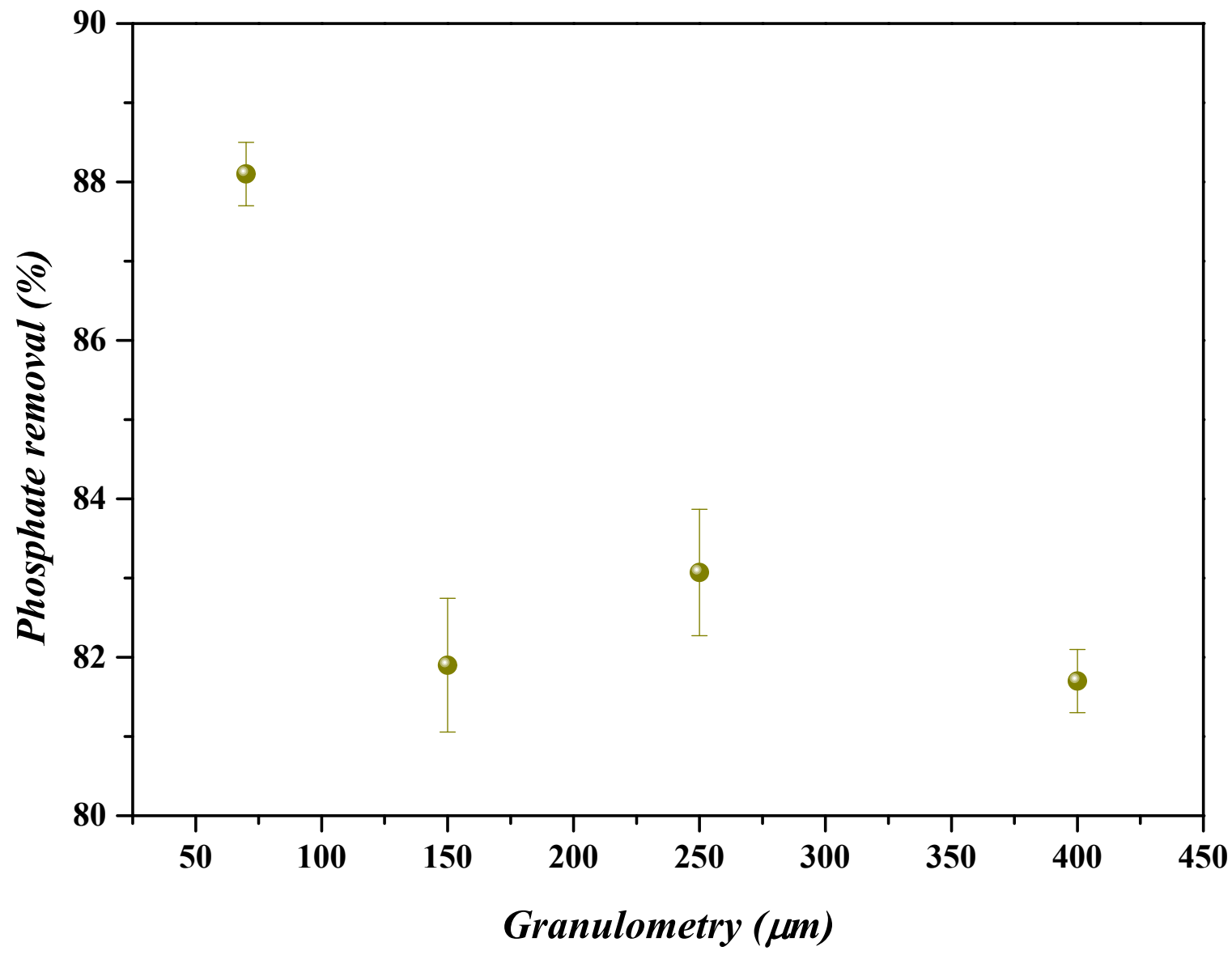

Table S4: Results of adsorption kinetics modeling. Adsorption parameters: BiMag dosage = 10 g/L; pH = 4; stirring = 150 rpm; temperature = 30°C; BiMag granulometry = 70 µm.

*	Dosage (g/L)	Phosphate conc. (mg/L)		PFO				PSO				Elovich			
			q_{e_exp} (mg/g)	q_e (mg/g)	k_1 (1/min)	R_{adj}^2	RMSE	q_e (mg/g)	k_2 (g/mg.min)	R_{adj}^2	RMSE	α (mg/g.min)	β (mg/g)	R_{adj}^2	RMSE
BiMag	10	10	0.80 ± 0.05	0.75 ± 0.02	0.29 ± 0.05	0.9663	0.0406	0.77 ± 0.01	0.76 ± 0.15	0.9876	0.0246	2837.04 ± 599.52	20.78 ± 0.31	0.9999	0.0025
	10	50	4.56 ± 1.21	3.80 ± 0.29	0.11 ± 0.04	0.7785	0.5968	4.17 ± 0.28	0.03 ± 0.01	0.8783	0.4424	2.50 ± 1.35	1.62 ± 0.18	0.9669	0.2307
	10	100	9.59 ± 2.69	9.30 ± 0.25	0.04 ± 0.005	0.9800	0.4463	10.28 ± 0.22	0.005 ± 6.1E-4	0.9924	0.2750	1.28 ± 0.39	0.52 ± 0.05	0.9769	0.4788

Table S5: Results of adsorption isotherms modeling. Adsorption parameters: BiMag dosage = 10 g/L; pH = 4; contact time = 15h; stirring = 150 rpm; BiMag granulometry = 70 µm.

*	T (°C)	q_{m_exp} (mg/g)	Langmuir q_m (mg/g)	Langmuir k_L (L/mg)	Langmuir R_{adj}^2	Langmuir RMSE	Freundlich k_F (mg g-1 (mg L-1)-1/n)	Freundlich n	Freundlich R_{adj}^2	Freundlich RMSE
BiMag	25	93.64 ± 2.87	138.63 ± 13.16	0.03 ± 0.006	0.9743	4.2851	8.50 ± 1.79	1.7 ± 0.17	0.9529	5.8014
BiMag	30	94.55 ± 2.89	132.93 ± 11.38	0.05 ± 0.009	0.9699	4.6991	11.71 ± 2.59	1.84 ± 0.22	0.9221	7.5636
BiMag	35	96.23 ± 2.92	129.05 ± 11.10	0.08 ± 0.02	0.9622	5.3476	15.82 ± 3.23	1.93 ± 0.25	0.9047	8.4957
BiMag	40	97.24 ± 2.94	136.31 ± 15.11	0.09 ± 0.02	0.9479	6.3334	16.33 ± 3.37	1.81 ± 0.24	0.9006	8.7486

*	T (°C)	q_{m_exp} (mg/g)	Sips q_m (mg/g)	Sips k_S (L/mg)	Sips n	Sips R_{adj}^2	Sips RMSE	Dubinin-Radushkevich q_m (mg/g)	Dubinin-Radushkevich kDR (mol²/kJ²)	Dubinin-Radushkevich E (kJ/mol)	Dubinin-Radushkevich R_{adj}^2	Dubinin-Radushkevich RMSE
BiMag	25	93.64 ± 2.87	122.79 ± 26.78	0.03 ± 0.01	1.11 ± 0.24	0.9716	4.2144	80.38 ± 4.45	1.37E-5 ± 2.64E-6	191.04	0.9137	7.8485
BiMag	30	94.55 ± 2.89	102.55 ± 9.44	0.03 ± 0.01	1.42 ± 0.23	0.9778	3.7741	83.70 ± 3.86	8.27E-6 ± 1.23E-6	245.88	0.9420	6.5250
BiMag	35	96.23 ± 2.92	99.91 ± 7.76	0.05 ± 0.02	1.49 ± 0.24	0.9747	4.0943	84.44 ± 4.10	3.30E-6 ± 5.38E-7	389.25	0.9361	6.9561
BiMag	40	97.24 ± 2.94	96.92 ± 7.76	0.05 ± 0.02	1.69 ± 0.32	0.9658	4.7981	86.91 ± 3.54	2.47E-6 ± 3.09E-7	449.92	0.9558	5.8321

Table S6: Adsorption thermodynamics parameters.

Composite	T (°C)	ΔG° (kJ/mol)	ΔH° (kJ/mol)	ΔS° (kJ/mol)	R_{adj}^2	RMSE
BiMag	25	-46.11	49.30 ± 13.14	0.32 ± 0.04	0.8738	3.54E7
	30	-47.71				
	35	-49.31				
	40	-50.91				

Table S7: Efficiency of adsorption-desorption cycles. Adsorption parameters: BiMag dosage = 10 g/L; pH in adsorption process = 4; contact time = 360min; stirring = 150 rpm; BiMag granulometry = 70 µm.

*	Adsorption-Desorption cycles											
	1° Cycle			2° Cycle			3° Cycle			4° Cycle		
Eluent	q_e (mg/g)	q_{des} (mg/g)	Regeneration (%)	q_e (mg/g)	q_{des} (mg/g)	Regeneration (%)	q_e (mg/g)	q_{des} (mg/g)	Regeneration (%)	q_e (mg/g)	q_{des} (mg/g)	Regeneration (%)
NaOH 0.1M	0.86	0.83	96.55	0.51	0.47	92.16	0.57	0.36	63.95	0.29	0.13	43.86
NaOH 0.5M	0.84	0.81	95.89	0.55	0.36	65.45	0.55	0.25	45.45	0.04	0.01	32.14
NaOH 1M	0.83	0.79	95.74	0.47	0.24	50.60	0.56	0.22	39.29	0.10	0.02	24.04
